# PIF4 Coordinates Thermosensory Growth and Immunity in *Arabidopsis*

**DOI:** 10.1016/j.cub.2016.11.012

**Published:** 2017-01-23

**Authors:** Sreeramaiah N. Gangappa, Souha Berriri, S. Vinod Kumar

**Affiliations:** 1 Cell and Developmental Biology Department, John Innes Centre, Norwich NR4 7UH, UK

**Keywords:** PIF4, PHYB, thermosensory growth, immunity, growth-defense trade-off, environmental signal integration, natural variation, adaptation, temperature resilience

## Abstract

Temperature is a key seasonal signal that shapes plant growth. Elevated ambient temperature accelerates growth and developmental transitions [1] while compromising plant defenses, leading to increased susceptibility [2, 3]. Suppression of immunity at elevated temperature is at the interface of trade-off between growth and defense [2, 4]. Climate change and the increase in average growth-season temperatures threaten biodiversity and food security [5, 6]. Despite its significance, the molecular mechanisms that link thermosensory growth and defense responses are not known. Here we show that PHYTOCHROME INTERACTING FACTOR 4 (PIF4)-mediated thermosensory growth and architecture adaptations are directly linked to suppression of immunity at elevated temperature. PIF4 positively regulates growth and development and negatively regulates immunity. We also show that natural variation of PIF4-mediated temperature response underlies variation in the balance between growth and defense among *Arabidopsis* natural strains. Importantly, we find that modulation of PIF4 function alters temperature sensitivity of defense. Perturbation of PIF4-mediated growth has resulted in temperature-resilient disease resistance. This study reveals a molecular link between thermosensory growth and immunity in plants. Elucidation of the molecular mechanisms that define environmental signal integration is key to the development of novel strategies for breeding temperature-resilient disease resistance in crops.

## Highlights

• Transcription factor PIF4 coordinates thermosensory growth and immunity
• PIF4 negatively regulates immunity, while promoting growth
• Natural variation of PIF4 signaling underlies growth-defense balance in the wild
• Modulation of PIF4 signaling alters temperature sensitivity of disease resistance

## Graphical Abstract

**Figure:**
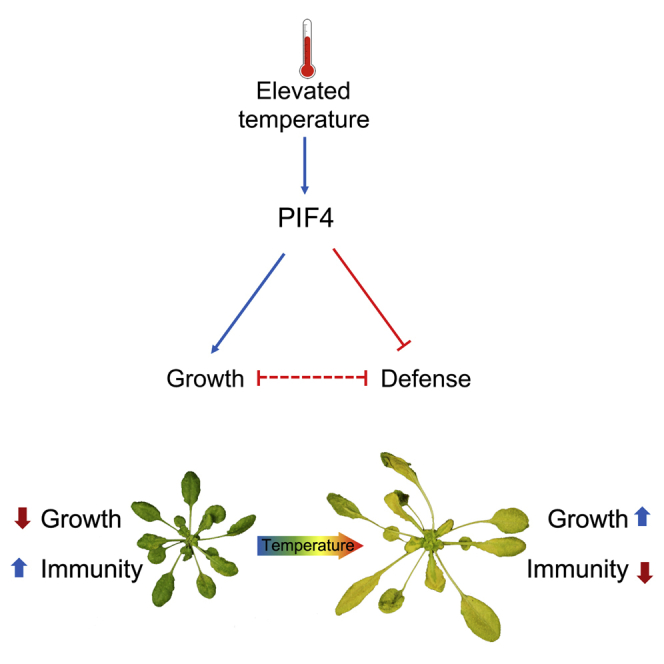

## Teaser

Gangappa et al. show that the transcription factor PIF4 coordinates thermosensory growth and immunity. PIF4 acts as a negative regulator of plant immunity, and modulation of its function alters the balance between growth and defense. Importantly, natural variation of PIF4 signaling underlies growth-defense balance in *Arabidopsis* natural strains.

## Results and Discussion

### PIF4 Signaling Is Required for Temperature-Induced Suppression of the *snc1-1* Phenotype

**Figure 1:**
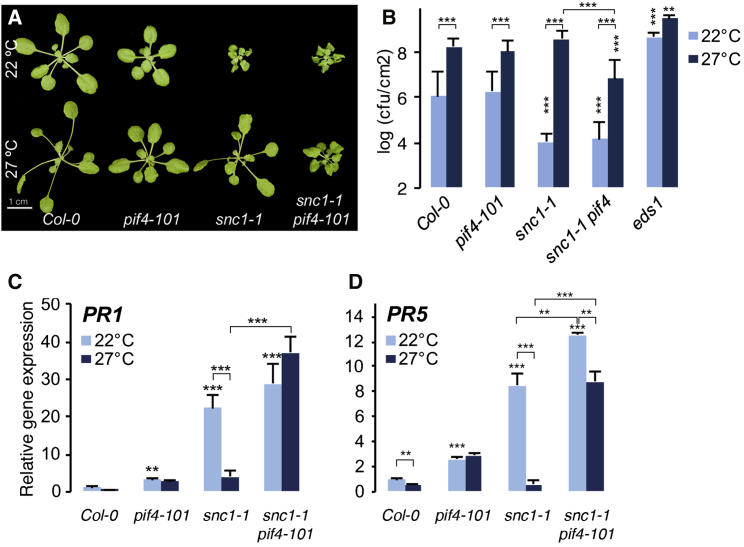
PIF4 Is Essential for the Suppression of Immunity by Elevated Temperature Analysis of *snc1-1 pif4-101* double mutants shows that temperature-induced suppression of *snc1-1* phenotypes is PIF4 dependent. (A) Morphological phenotypes of 4-week-old plants grown at 22°C and 27°C under a short-day photoperiod. (B) Temperature-induced suppression of disease resistance of *snc1-1* is PIF4 dependent. Resistance phenotype of the indicated genotypes to *P. syringae pv*. tomato (*Pto*) DC3000 (A_600_ 0.02) at 22°C and 27°C (mean ± SD; n ≥ 8). ^∗∗^p ≤ 0.01, ^∗∗∗^p ≤ 0.001 (two-way ANOVA with Tukey’s multiple comparison test) compared to the corresponding Col-0 or as indicated; cfu, colony forming unit. (C and D) Gene expression analysis of defense marker genes *PR1* (C) and *PR5* (D) by qRT-PCR (mean ± SD of three biological replicates) from 3-week-old plants. ^∗∗^p ≤ 0.01, ^∗∗∗^p ≤ 0.001 (two-way ANOVA with Tukey’s multiple comparison test) significantly different from either Col-0 or between the indicated pairs. See also Figure S1.

Elevated temperature in spring promotes growth and accelerates developmental transitions [1], whereas it strongly suppresses defense responses. Trade-off between growth and immunity underlies the compromised resistance at higher temperatures [2]. One of the well-studied examples of temperature modulation of immunity is the suppression of resistance mediated by nucleotide-binding and leucine-rich repeat (NB-LRR) proteins such as SNC1 (SUPPRESSOR OF npr1-1, CONSTITUTIVE 1). The *snc1-1* mutation leads to constitutive activation of defense responses and severe growth defects [7], both of which are completely suppressed at higher ambient temperature [8]. The molecular mechanisms underlying immunity suppression by elevated temperature are not well understood. PHYTOCHROME INTERACTING FACTOR 4 (PIF4), a basic-helix-loop-helix (bHLH) transcription factor, controls thermosensory growth and architecture adaptations as well as reproductive transition in *Arabidopsis* [9, 10] and functions as an integrator of environmental cues [11, 12]. To test whether PIF4-mediated thermosensory signaling is involved in the modulation of immunity at elevated temperature, we studied the suppression of SNC1-mediated defense responses in the *snc1-1 pif4-101* double mutant. Growth defects of *snc1-1*, not the *snc1-1 pif4-101* double mutant, were suppressed by growth at 27°C (Figure 1A; Figures S1A–S1C). Increased resistance to *Pseudomonas syringae* pv. tomato (*Pto*) DC3000 of *snc1-1*, not *snc1-1 pif4-101*, is suppressed to wild-type levels at 27°C (Figure 1B). Further, gene expression analyses by qRT-PCR analysis of *PR1* and *PR5* (Figures 1C and 1D) have confirmed that the temperature-induced suppression of constitutively expressed defense genes in *snc1-1* was also *PIF4* dependent. Taken together, these results show that PIF4-mediated thermosensory signaling plays an important role in the suppression of defense by elevated temperature.

### PIF4 Is a Negative Regulator of Immunity

**Figure 2:**
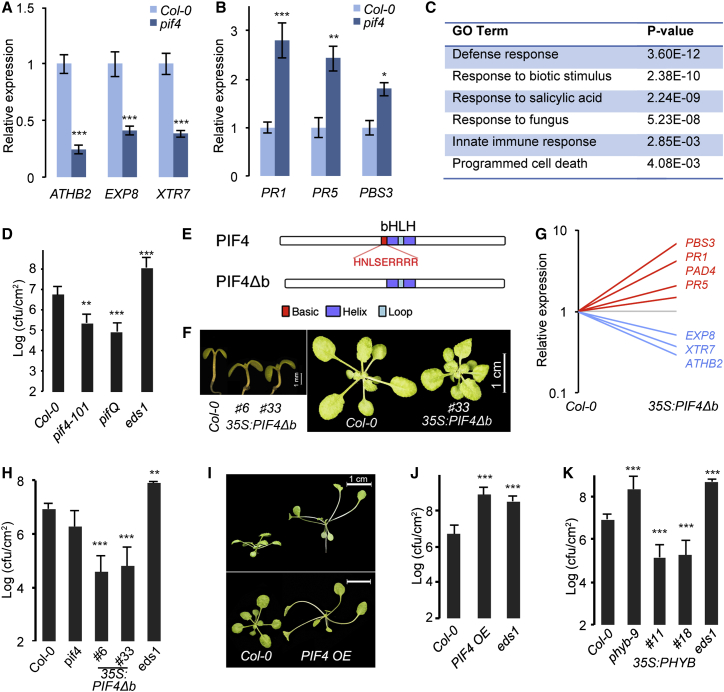
PIF4 Is a Negative Regulator of Immunity (A and B) Downregulation of growth-related genes (A) and upregulation of defense genes (B) in *pif4-101* as shown by qRT-PCR (mean ± SD of three biological replicates) from 1-week-old seedlings grown at 22°C under a short-day photoperiod. (C) Enrichment of defense GO terms in genes upregulated in 1-week-old *pif4-101* (Dataset S1) seedlings grown at 22°C under a short-day photoperiod. (D) Increased disease resistance of *pif4-101* and *pifQ* mutants to *Pto* DC3000 (A_600_ 0.002; mean ± SD; n = 8). (E) Schematic representation of PIF4Δb, which lacks the basic domain. (F) Reduced hypocotyl elongation growth and rosette phenotype in two independent lines overexpressing *PIF4Δb*. (G) Overexpression of *PIF4Δb* leads to downregulation of growth (blue) and upregulation of defense (red) genes (data are the average of three biological replicates; see also Figure S1) in 22°C short-day-grown seedlings for 1 week. (H) Disease-resistance phenotype of 35S:*PIF4*Δ*b* to *Pto* DC3000 (A_600_ 0.02; mean ± SD; n ≥ 12). (I) *PIF4-FLAG* OE showing enhanced elongation growth. (J and K) Disease-resistance phenotype of *PIF4-FLAG* OE (J) and *35S*:*PHYB*-*FLAG* (K) lines to *Pto* DC3000 (A_600_ 0.02; mean ± SD; n ≥ 12). ^∗^p ≤ 0.05, ^∗∗^p ≤ 0.01, ^∗∗∗^p ≤ 0.001 (Student’s t test) significantly different from Col-0. In (D), (H), (J), and (K), plants grown at 22°C under a short-day photoperiod for 4 weeks were used for the resistance assays. See also Figure S1.

The above results led us to hypothesize that PIF4 signaling could modulate defense responses. Gene expression analysis by qRT-PCR on 7-day-old seedlings grown at 22°C showed that whereas the expression of PIF4 target genes related to growth such as *ATHB2*, *EXP8*, and *XTR7* were downregulated in the *pif4-101* mutant as expected (Figure 2A), defense-related genes such as *PR1*, *PR5*, and *PBS3* were upregulated (Figure 2B), showing that PIF4 modulates immunity in *Arabidopsis*. Furthermore, RNA-sequencing (RNA-seq) analysis showed that genes that are upregulated in *pif4-101* were significantly enriched for defense-related Gene Ontology (GO) terms (Figure 2C; Data S1). Accordingly, the *pif4-101* and *pifQ* (*pif1 pif3 pif4 pif5*) quadruple mutant [13] showed increased resistance to *Pto* DC3000 when challenged with a lower inoculum (A_600_ 0.002) (Figure 2D) but not significantly when a higher bacterial titer (A_600_ 0.02) was used (Figure S1D). Together, the modulation of defense gene expression and alteration of disease resistance in the mutants show that PIF4 acts as a negative regulator of immunity.

PIF4, a bHLH transcription factor [14], functions cooperatively with other PIFs [11, 15] as well as with other proteins involved in growth and immunity [12, 16, 17]. The modest increase in resistance in *pif4* and *pifQ* could be reflecting the quantitative contribution of these, including PIF7 [14], to defense modulation. The bHLH transcription factors function as hetero- or homo-dimers and require the basic (b) domain for DNA binding [14]. Dimerization with a protein lacking the basic domain renders them non-DNA binding and therefore non-functional [18, 19]. Therefore, with the aim of producing a dominant negative with little or no PIF function, we generated a PIF4 variant lacking the basic domain, hereafter referred to as PIF4Δb (Figure 2E). When overexpressed, *PIF4Δb* resulted in strong suppression of growth (Figure 2F; Figures S1E and S1F), suggesting that PIF4Δb acts as a dominant negative as expected. Further substantiating this, PIF4Δb strongly suppressed the enhanced growth promoted by 35S:*PIF4-HA* (hemagglutinin) (Figure S1G). Consistent with this, *35S:PIF4Δb* led to downregulation of growth-related genes and upregulation of defense genes (Figure 2G; Figures S1H and S1I) and enhanced resistance to *Pto* DC3000 (Figure 2H), further substantiating the role of PIF4 in modulating immunity. In a complementary experiment, we analyzed a *P*_*PIF4*_*:PIF4-FLAG* transgenic line showing *PIF4* overexpression (*PIF4-OE*) (Figure S1J) that showed enhanced elongation growth (Figure 2I; Figure S1K). Interestingly, *PIF4-OE* showed increased expression of growth-related genes and reduced defense gene expression (Figures S1L and S1M) as well as increased susceptibility to *Pto* DC3000 (Figure 2J), showing that PIF4 is sufficient to modulate immunity.

The photoreceptor phytochrome B (PHYB) regulates PIF transcription factors at the protein level through promoting light-dependent protein degradation. Loss-of-function *phyb* mutants show exaggerated PIF-mediated growth [11, 20, 21]. Supporting our above results and consistent with earlier reports [22], *phyb*-9 showed increased susceptibility to *Pto* DC3000 (Figure 2K). Conversely, *35S:PHYB-FLAG* transgenic lines (Figure S1N) showed reduced growth (Figure S1O) concomitant with decreased expression of growth-related genes (Figure S1P). In line with the role of the PIF-PHYB module in growth-defense balance, the *35S:PHYB-FLAG* lines showed increased defense gene expression (Figures S1Q and S1R) and enhanced resistance to *Pto* DC3000 (Figure 2K). These results further established the role of PIF4 signaling in coordinating plant growth and immunity.

### Natural Variation in PIF4 Signaling Underlies Growth-Defense Balance

**Figure 3:**
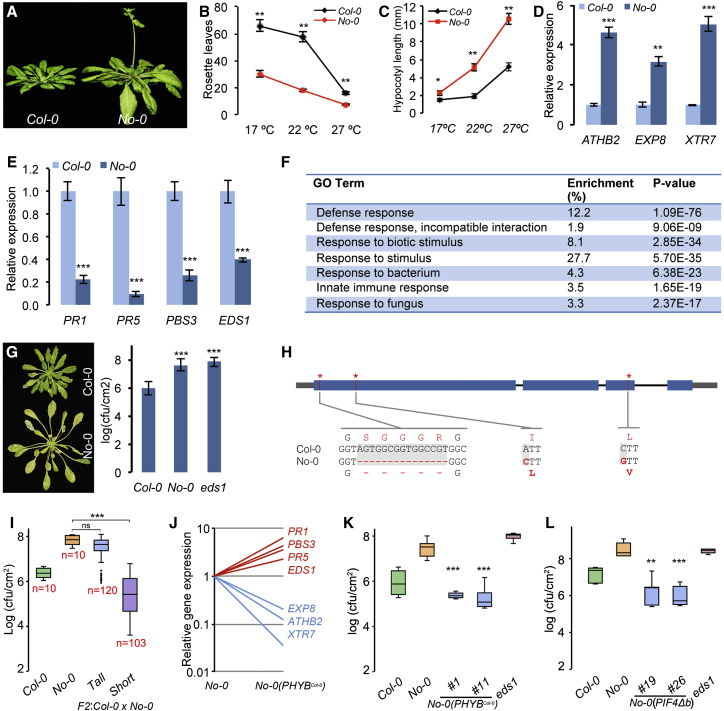
Natural Variation of PIF4-Mediated Thermosensory Growth and Immunity (A–C) No-0 shows robust growth (A) and enhanced thermosensory flowering (B) and hypocotyl growth (C). (D and E) Gene expression of growth (D) and defense markers (E) in No-0 as shown by qRT-PCR (mean ± SD of three biological replicates). (F) GO analysis showing that genes downregulated in No-0 (see also Data S2) are enriched for defense GO terms. (G) No-0 shows increased susceptibility to *Pto* DC3000 (A_600_ 0.02; mean ± SD; n ≥ 10). (H) Diagrammatic representation of *PHYB*^*No-0*^ showing polymorphisms at the nucleotide and amino acid level. (I) Resistance to *Pto* DC3000 (A_600_ 0.02) of F_2_ segregants of a Col-0 × No-0 cross showing co-segregation of growth and defense phenotypes. Three-week-old plants grown in 22°C under a short-day photoperiod were used for the experiment. (J) Transgenic expression of *PHYB:PHYB*^*Col-0*^ fully complements gene expression phenotypes of No-0 (mean of three biological replicates; see also Figure S2). (K) Disease-resistance phenotypes of two independent transgenic lines of No-0 complemented with *PHYB:PHYB*^*Col-0*^. (L) Overexpression of *PIF4*Δ*b* in No-0 leads to increased resistance to *Pto* DC3000 (A_600_ 0.02) (two independent transgenic lines are shown). In (C)–(E) and (J), 1-week-old seedlings grown at 22°C under a short-day photoperiod were used for the experiments. In (G), (K), and (L), 4-week-old plants grown at 22°C under a short-day photoperiod were used for the resistance assays. ^∗^p ≤ 0.05, ^∗∗^p ≤ 0.01, ^∗∗∗^p ≤ 0.001 (Student’s t test) significantly different from either Col-0 (in B–E and G) or No-0 (in I, K, and L). See also Figures S2 and S3.

In nature, growth and development are fine-tuned to suit the prevailing local environmental conditions [23, 24]. We examined natural variation of thermosensory growth in *Arabidopsis* in relation to defense. The natural accession Nossen (*No-0*) showed robust growth (Figure 3A), enhanced thermosensory flowering (Figure 3B), and enhanced temperature-induced hypocotyl elongation (Figure 3C) under a short-day photoperiod, phenocopying Columbia (*Col*)*-0* plants grown at warmer temperatures. Consistent with this, expression of growth-related genes was significantly upregulated in *No-0* (Figure 3D). This was accompanied by reduced expression of key defense marker genes (Figure 3E). Further, whole-genome transcriptome analysis by RNA-seq showed that the genes downregulated in *No-0* were enriched for defense-related GO terms (Figure 3F; Data S2). Accordingly, we found that *No-0* is more susceptible to *Pto* DC3000 (Figure 3G), suggesting that enhanced thermosensory growth in *No-0* leads to compromised basal immunity.

Growth and defense phenotypes of *No-0* were reminiscent of the *phyb*-9 mutant (Figures S2A–S2C). Moreover, F_1_ seedlings from a *No-0* × *phyb*-9 cross did not show complementation of the *phyb-9* phenotype (Figure S2D), suggesting that PHYB function could be compromised in *No-0*. *PHYB* expression in *No-0*, however, was comparable to *Col-0* (Figure S2E). To test whether the altered PHYB function is due to variation at the *PHYB* locus, we carried out a comparative sequence analysis. We found that the *PHYB* locus of *No-0*, hereafter referred to as *PHYB*^*No-0*^, is polymorphic. *PHYB*^*No-0*^ has a 15 bp deletion, causing an in-frame deletion (ΔSGGGR) at the N terminus, and two non-synonymous SNPs leading to amino acid substitutions I143L and L1072V (Figure 3H), which were previously shown to be associated with PHYB function [25]. Interestingly, hierarchical clustering of *Arabidopsis* natural accessions and mutants for light responses resulted in No-0 and *phyb* alleles, particularly *phyb-9*, to cluster together [26], providing further evidence that No-0 is perturbed in PHYB function.

To test whether the variant *PHYB*^*No-0*^ allele underlies the variation in growth and immunity, we analyzed the F_2_ population of a cross between *Col-0* and *No-0* for growth and defense phenotypes (Figure 3I). Long-hypocotyl and disease-susceptibility phenotypes were strongly associated with *PHYB*^*No-0*^, whereas short hypocotyl and disease resistance were associated with *PHYB*^*Col-0*^ (Figure 3I; Figure S2F), suggesting that PHYB is the major determinant of phenotypic variation. It is also possible that other factors such as *PIF4* itself could add to *PHYB* in balancing growth and defense in No-0. Further, *P*_*PHYB*_:*PHYB*^*Col-0*^ fully complemented the growth and gene expression phenotypes of *No-0* (Figure 3J; Figures S2G–S2J). Moreover, the complemented lines showed increased resistance to *Pto* DC3000, which was comparable to *Col-0* (Figure 3K). Together, these results confirmed that the hypomorphic *PHYB*^*No-0*^ underlies the altered growth-defense balance.

Because PHYB negatively regulates PIF4, we hypothesized that the phenotypes of *No-0* could be due to enhanced PIF4 function. Interestingly, *No-0* also showed increased expression of *PIF4* (Figure S3A). Moreover, overexpression of the dominant-negative PIF4Δb (Figure S3B) strongly suppressed the growth phenotypes (Figures S3C and S3D). PIF4Δb transgenic lines showed reduced expression of growth-related genes (Figure S3E), while enhancing defense gene expression (Figure S3F) and disease resistance (Figure 3L). We therefore conclude that the altered growth-defense balance in *No-0* is due to increased PIF4 function, as a result of reduced PHYB-mediated repression. It could also be at least in part due to increased *PIF4* expression (Figure S3A).

To test whether *PHYB* allelic variation is reflected in altered growth-immunity balance more widely in nature, we analyzed the worldwide set of 96 *Arabidopsis* natural accessions [27]. Comparative analysis of deduced PHYB amino acid sequences (Figure S3G) identified Edinburgh (*Edi*)*-0*, Kashmir (*Kas*)*-1*, and Shakdara (*Sha*) to have similar PHYB protein as *No-0*, including the I143L and L1072V substitutions (Figure S3H). All three accessions showed enhanced elongation of hypocotyl (Figures S3I–S3K) and robust growth (Figure S3N) phenocopying *No-0*, which was accompanied by upregulated expression of growth-related genes (Figure S3L) and downregulation of defense-related genes (Figure S3M). Most importantly, these accessions showed increased susceptibility to *Pto* DC3000 (Figures S3O and S3P), confirming that the PHY-PIF signaling module coordinates growth and defense in the wild.

### Modulation of PIF4 Signaling Alters Temperature-Induced Modulation of Immunity

**Figure 4:**
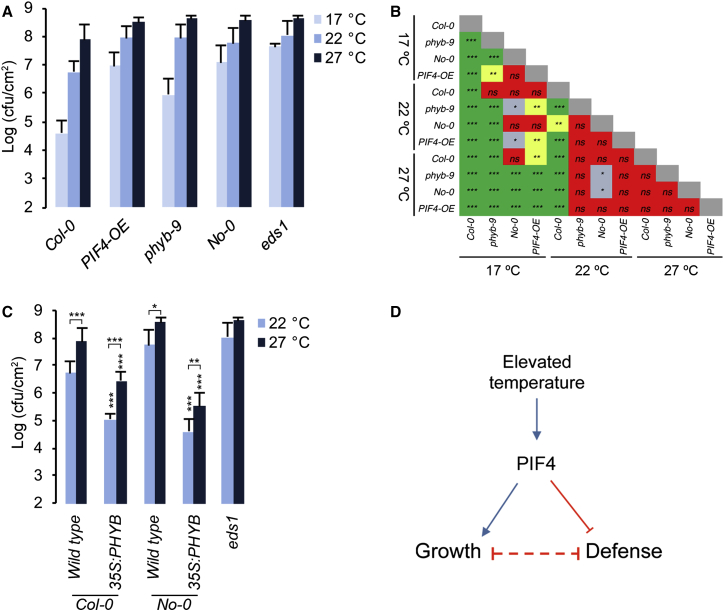
PIF4-Mediated Thermosensory Signaling Modulates Temperature Sensitivity of Immunity (A) Increased PIF4 function (in *PIF4-OE*, *phyb-9*, and No-0) leads to increased susceptibility to *Pto* DC3000 (A_600_ 0.002) at lower temperatures, phenocopying wild-type plants grown at higher temperature. (B) Two-way ANOVA analysis with Tukey’s multiple comparison test of data from (A); ^∗^p ≤ 0.05, ^∗∗^p ≤ 0.01, ^∗∗∗^p ≤ 0.001; ns, not significant. (C) *PHYB* overexpression leads to temperature-resilient disease resistance to *Pto* DC3000 (A_600_ 0.002) (mean ± SD; n = 8). ^∗^p ≤ 0.05, ^∗∗^p ≤ 0.01, ^∗∗∗^p ≤ 0.001 (two-way ANOVA with Tukey’s multiple comparison test) significantly different from either Col-0 at respective temperatures or between the indicated pairs. See also Figure S4. (D) Model showing PIF4 function at the interface of growth and defense responses. While promoting thermosensory growth, PIF4 negatively regulates immunity. See also Figure S4.

The above results have clearly shown that PIF4 coordinates growth and defense responses. To test whether PIF4 signaling also controls temperature sensitivity of defense, we studied disease resistance at elevated temperature. We tested whether increased PIF4 signaling could lead to increased susceptibility at lower temperature. When grown at 17°C, wild-type Col-0 shows increased resistance to *Pto* DC3000 (Figures 4A and 4B). Further substantiating the role of PIF4 in modulating defense, *PIF4-OE* showed increased susceptibility at 17°C and 22°C, phenocopying Col-0 plants grown at 22°C and 27°C, respectively (Figures 4A and 4B), showing that PIF4 is sufficient for mediating temperature-induced susceptibility. In line with this, *phyb-9* and *No-0* showed strongly reduced resistance to *Pto* DC3000 at lower temperature, phenocopying growth at elevated temperatures (Figures 4A and 4B). Consistent with the established signaling hierarchy, *PIF4-OE* showed reduced temperature sensitivity of resistance compared to *phyb-9*. Further, the *35S:PIF4*Δ*b* line showed significantly increased resistance even at 27°C compared to Col-0 (Figure S4A). Conversely, *35S:PHYB-GFP* [28] strongly enhanced *snc1*-*1* phenotypes at 22°C and prevented its suppression at 27°C (Figures S4B–S4D). Accordingly, a transgenic line overexpressing *PHYB*^*Col-0*^ in *No-0* [29] strongly suppressed growth (Figures S4E–S4G), and has resulted in enhanced defense gene expression even at 27°C (Figures S4H–S4J). Importantly, this has resulted in temperature-resilient resistance to *Pto* DC3000 at 27°C (Figure 4C). Together, our data clearly show that the PIF4-mediated thermosensory signaling module is both essential and sufficient to modulate temperature sensitivity of defense responses.

### Conclusions

Collectively, our data show that PIF4, a central component of temperature responses, coordinates thermosensory growth and immunity (Figure 4D). Natural variation of PIF4-mediated growth defines the balance between growth and immunity in the wild. To grow robustly and to effectively fend off pathogens are extremely desirable traits. However, trade-offs between these processes lead to optimization of growth and defense in nature, exemplified by strategies where reduced growth leads to a fitness advantage of being well protected from pathogens [24]. Similarly, robust growth at the cost of reduced defense could be beneficial when pathogen load is low or under conditions that restrict growth. Lower temperature and insufficient resources such as nutrients and light quality due to competition or seasonal fluctuations are growth limiting. PHYB is a major regulator that limits growth in response to the environment. Therefore, accessions such as *No-0* could have a fitness advantage through robust growth and shorter life cycle that could help evade pathogens. Conversely, enhanced PHYB-mediated growth restraint may be advantageous under warmer environments, especially in the context of climate change [5, 6]. Being a central environmental signaling hub, PIF4 could therefore be involved in coordinating growth and defense in response to a number of environmental signals including light quality and during shade-avoidance responses. Understanding the mechanistic framework of environmental signal integration will be vital for breeding climate-resilient crops. This study unravels such a mechanism whereby growth and defense responses are coordinated in response to the environment.

## Author Contributions

S.N.G. designed and performed most of the experiments and analyzed data. S.B. contributed to the experiments and data analysis. S.V.K. designed and supervised the study and analyzed data. S.N.G. and S.V.K. wrote the paper.

## Supplemental Information

Document S1. Supplemental Experimental Procedures and Figures S1–S4

Data S1. Gene Expression Analysis of *pif4-101* Mutant

Data S2. Gene Expression Analysis of *No-0*

Document S2. Article plus Supplemental Information
