# Supplementary material for: PIF4 Coordinates Thermosensory Growth and Immunity in Arabidopsis
Source: Curr Biol. 2017 Jan 23;27(2):243–9. doi: 10.1016/j.cub.2016.11.012 (PMC5266789; doi:10.1016/j.cub.2016.11.012)
Supplement: Document S1. Supplemental Experimental Procedures and Figures S1–S4 [file mmc1.pdf]

Current Biology, Volume *27*

Supplemental Information

PIF4 Coordinates Thermosensory Growth and Immunity in *Arabidopsis*

Sreeramaiah N. Gangappa, Souha Berriri, and S. Vinod Kumar

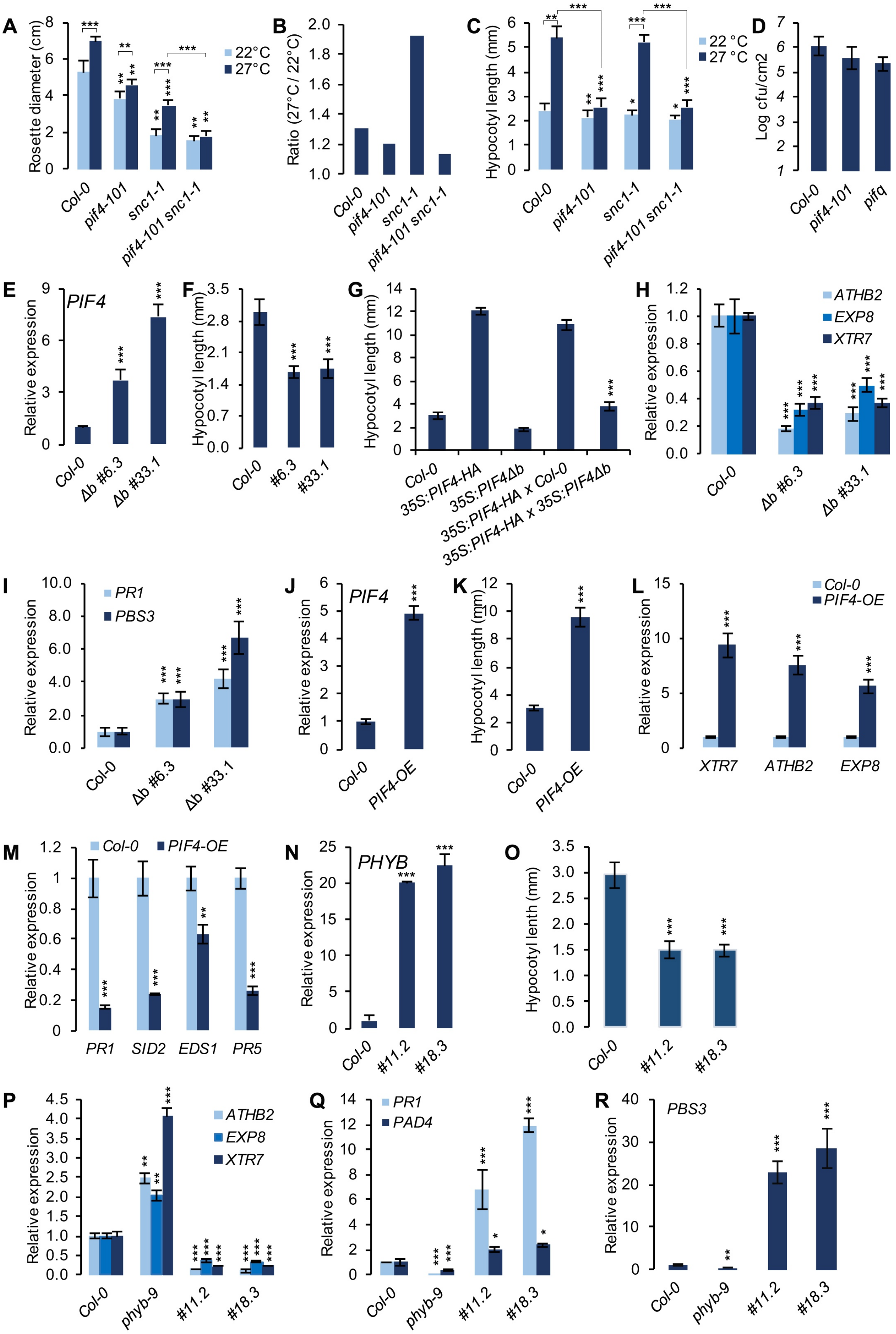

A
Rosette diameter (cm)
22°C
27°C
Col-0
pif4-101
snc1-1
pif4-101 snc1-1
B
Ratio (27°C / 22°C)
C
Hypocotyl length (mm)
22 °C
27 °C
D
Log cfu/cm2
pifq
E
PIF4
Relative expression
Δb #6.3
Δb #33.1
F
Hypocotyl length (mm)
#6.3
#33.1
G
Hypocotyl length (mm)
35S:PIF4-HA
35S:PIF4Δb
35S:PIF4-HA x Col-0
35S:PIF4-HA x 35S:PIF4Δb
H
Relative expression
ATHB2
EXP8
XTR7
I
Relative expression
PR1
PBS3
J
PIF4
Relative expression
PIF4-OE
K
Hypocotyl length (mm)
L
Relative expression
Col-0
PIF4-OE
M
Relative expression
PR1
SID2
EDS1
PR5
N
PHYB
Relative expression
#11.2
#18.3
O
Hypocotyl lenth (mm)
P
Relative expression
phyb-9
Q
Relative expression
PAD4
R
PBS3
Relative expression

Figure S1. PIF4 negatively regulates immunity (Related to Figure1 and Figure 2)

(**A-C**) PIF4 is essential for the elevated temperature mediated suppression of *snc1-1* growth phenotypes. Quantification of rosette diameter (A; mean±SD, n≥8), the response ratio (27°C/22°C; B), and hypocotyl elongation (**C;** mean±SD, n≥20). For the measurement of rosette diameter, plants grown at 22°C under short-day photoperiod for three-weeks were either retained at 22°C or exposed to 27°C for 6-days. On day 6 plants were photographed and diameter was measured using ImageJ software. (**D**) Resistance phenotype of *pif4-101* and *pifq* (*pif1 pif3 pif4 pif5*) mutants to *Pto* DC3000 (A_{600}=0.02; Mean±SD; n=6). Four-week-old plants grown at SD were used for the assay. (**E**) Expression of total *PIF4* (*PIF4* + *PIF4Δb*) in two independent *35S:PIF4Δb-Myc* (*35S:PIF4Δb*) lines (#6.3 and 33.1) as measured by qRT-PCR (mean±SD of three biological replicates). (**F**) Quantification of hypocotyl elongation (mean±SD; n≥20) in *35S:PIF4Δb* transgenic lines. (**G**) Overexpression of *PIF4Δb* strongly suppresses enhanced growth phenotypes of *35S:PIF4-HA.* Quantification of hypocotyl elongation (mean±SD; n≥20) of indicated genotypes. (**H** and **I**) Overexpression of *PIF4Δb-Myc* (35S:*PIF4Δb*) leads to downregulation of growth-related genes (**H**) and upregulation of defense responsive genes (**I**) as measured by qRT-PCR (mean±SD of three biological replicates). (**J**) Expression of *PIF4* in P_{PIF4}*:PIF4-FLAG* (*PIF4 OE*) transgenic line as measured by qRT-PCR (mean±SD of three biological replicates). (**K**) Quantification of hypocotyl elongation data (mean±SD; n≥20). (**L** and **M**) Increased expression of *PIF4* leads to upregulation of growth-related genes (**L**) and downregulation of defense (**M**) responsive genes (mean±SD of three biological replicates). (**N**) Expression of *PHYB* in two independent *35S:PHYB-FLAG (35S:PHYB)* transgenic lines (#11.2 and 18.3) as measured by qRT-PCR (mean±SD of three biological replicates). (**O**) Hypocotyl data (mean±SD; n≥20) of *35S:PHYB* seedlings. (**P-R**) Expression of growth (P) and defense responsive genes (Q and R) in *35S:PHYB* lines as measured by qRT-PCR (mean±SD of three biological replicates).

In Figure C and E-R, one-week-old seedlings grown at 22°C SD were used for the experiments. *P≤0.05, **P≤0.01, ***P≤0.001 (Student's *t*-test) significantly different from Col-0.

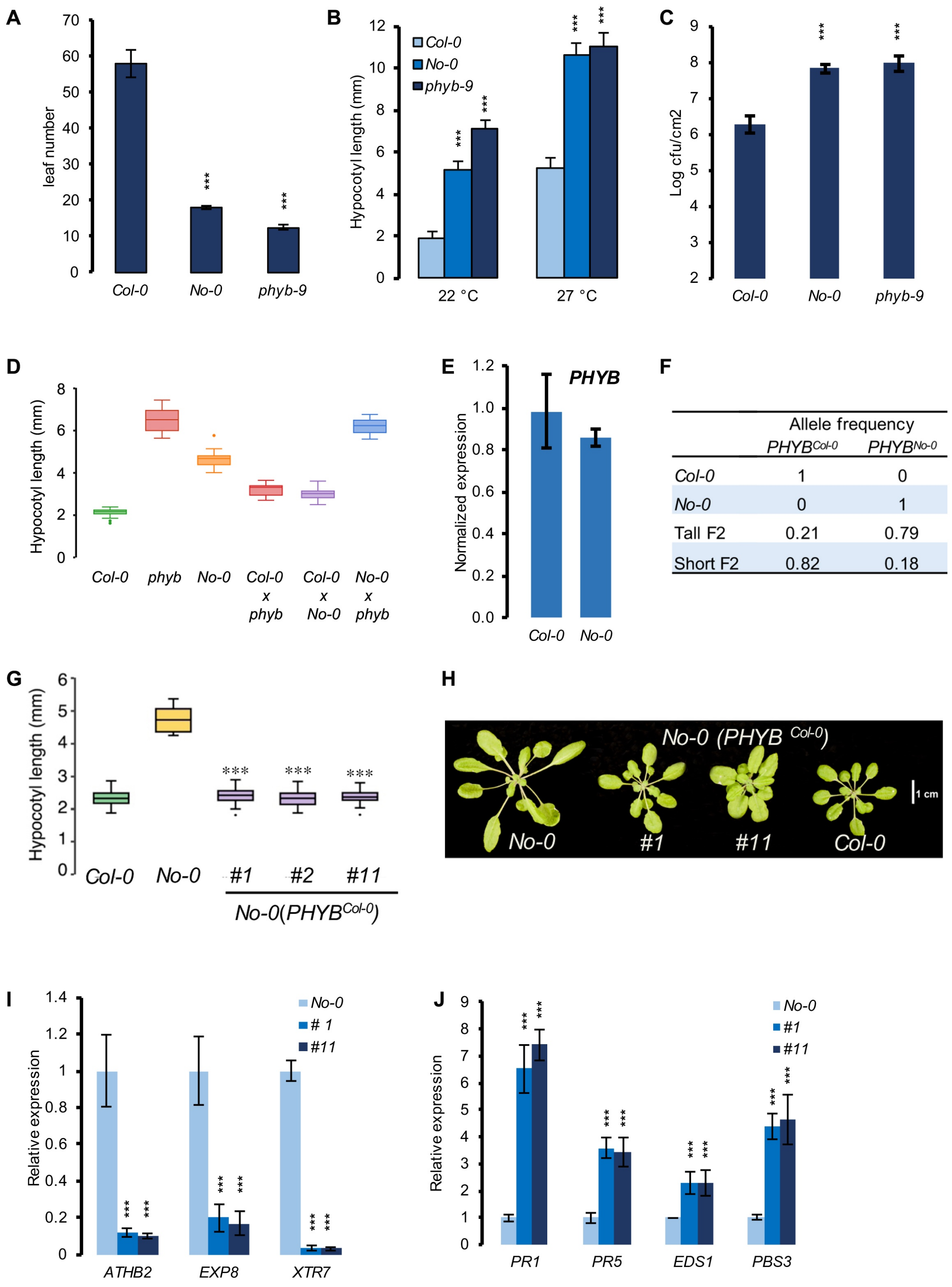

A
leaf number
Col-0
No-0
phyb-9
B
Hypocotyl length (mm)
Col-0
No-0
phyb-9
22 °C
27 °C
C
Log cfu/cm2
Col-0
No-0
phyb-9
D
Hypocotyl length (mm)
Col-0
phyb
No-0
Col-0 x phyb
Col-0 x No-0
No-0 x phyb
E
PHYB
Normalized expression
Col-0
No-0
F
Allele frequency
PHYBCol-0
PHYBNo-0
Col-0 1 0
No-0 0 1
Tall F2 0.21 0.79
Short F2 0.82 0.18
G
Hypocotyl length (mm)
Col-0
No-0
#1
#2
#11
No-0(PHYBCol-0)
H
No-0 (PHYB Col-0)
1 cm
No-0
#1
#11
Col-0
I
Relative expression
No-0
1
#11
ATHB2
EXP8
XTR7
J
Relative expression
No-0
#1
#11
PR1
PR5
EDS1
PBS3

Figure S2. ***PHYB* underlies altered growth-defense balance in No-0 (Related to Figure 3)**
(**A**) Quantification of the flowering time data (mean±SD; n≥8) showing No-0 has early flowering phenotype similar to *phyb-9*. (**B**) Quantification of hypocotyl elongation data (mean±SD; n≥20) indicating that No-0 phenocopying *phyb-9* at different temperatures. (**C**) No-0 is increasingly susceptible to *Pto* DC3000 (A_{600}=0.02) at 22°C similar to *phyb-9* (data is mean±SD; n≥10). (**D**) Hypocotyl data of 22°C grown one-week-old seedlings showing *No-0* is allelic to *phyb* as $PHYB^{No\text{-}0}$ cannot complement *phyb-9* hypocotyl phenotype (Data shown is mean±SD; n≥20). (**E**) Expression of *PHYB* in No-0 is not altered as measured by qRT-PCR (mean±SD of three biological replicates) in one-week-old seedlings grown at 22°C SD. (**F**) Segregation analysis of No-0 × Col-0 F_2 population showing Tall hypocotyl phenotype is strongly associated with $PHYB^{No\text{-}0}$ (allele frequency= 0.79*)* whereas short hypocotyl phenotype is strongly associated with $PHYB^{Col\text{-}0}$ (with allelic frequency of 0.82). (**G**) Quantification of hypocotyl length (mean±SD; n≥20) of one-week-old seedlings grown at 22° C SD. ***P≤0.001 (Student's *t*-test) significantly different from No-0. (**H**) Rosette picture of four-week-old adult plants grown in 22° C SD showing complementation of No-0 by $PHYB^{Col\text{-}0}$. (**I** and **J**) Expression of growth (I) and defense (J) responsive genes (mean±SD of three biological replicates) in No-0 and two independent complemented (P_{PHYB}:$PHYB^{Col\text{-}0}$) lines from one-week-old seedlings. **P≤0.01, ***P≤0.001 (Student's *t*-test) significantly different from No-0.

In Figure A-C and F, data shown is representative of at least two independent experiments. ***P≤0.001, (Student's *t*-test) significantly different from Col-0.

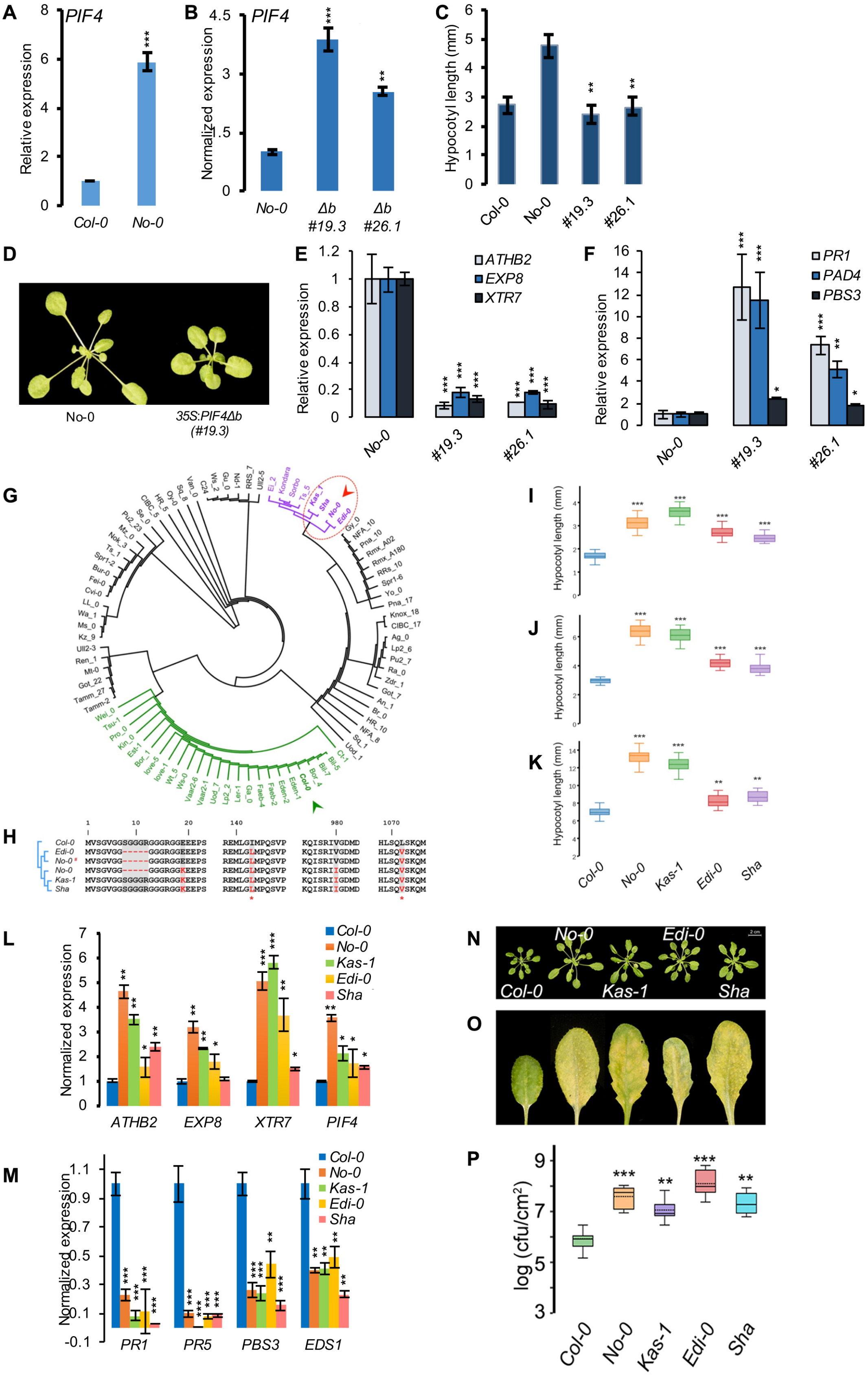
A
PIF4
Relative expression
Col-0
No-0
B
PIF4
Normalized expression
No-0
Δb #19.3
Δb #26.1
C
Hypocotyl length (mm)
Col-0
No-0
#19.3
#26.1
D
No-0
35S:PIF4Δb (#19.3)
E
ATHB2
EXP8
XTR7
Relative expression
No-0
#19.3
#26.1
F
PR1
PAD4
PBS3
Relative expression
No-0
#19.3
#26.1
G
H
Col-0
Edi-0
No-0
No-0
Kas-1
Sha
MVSGVGGSGGGRGGGRGGEEEPS
REMLGIMPQSVP
KQISRIVGDMD
HLSQLSKQM
I
J
K
Hypocotyl length (mm)
Col-0
No-0
Kas-1
Edi-0
Sha
L
Normalized expression
Col-0
No-0
Kas-1
Edi-0
Sha
ATHB2
EXP8
XTR7
PIF4
M
Normalized expression
Col-0
No-0
Kas-1
Edi-0
Sha
PR1
PR5
PBS3
EDS1
N
No-0
Edi-0
Col-0
Kas-1
Sha
O
P
log (cfu/cm²)
Col-0
No-0
Kas-1
Edi-0
Sha

Figure S3. Modulation of PIF4 function alters growth and defense in Arabidopsis natural accessions (Related to Figure 3)

(**A**) Expression of *PIF4* as measured by qRT-PCR (mean±SD of three biological replicates) in one-week-old seedlings grown at 22° C SD. ***P≤0.001 (Student's *t*-test) significantly different from Col-0 (**B**) Expression of total *PIF4* (*PIF4* + *PIF4Δb*) in two independent No-0(*35S:PIF4Δb-Myc*) transgenic lines (#19.3 and 26.1) as measured by qRT-PCR (mean±SD of three biological replicates). (**C**) Hypocotyl measurement data (mean±SD; n≥20) of No-0(*35S:PIF4Δb-Myc*) lines. (**D**) Rosette phenotype of No-0 overexpressing *PIF4Δb* showing reduced growth. (**E** and **F**) Expression of growth- (E) and defense responsive (F) genes as measured by qRT-PCR (mean±SD of three biological replicates). (**G**) Neighbour-joining tree of deduced PHYB amino acid sequence of the worldwide set of *Arabidopsis thaliana* natural accessions (obtained from 1001 genome project). Col-0 (green arrowhead) and No-0 (red arrowhead) are significantly divergent from each other. Edi-0, Kas-1, Sha and No-0 are highlighted to show their close similarity. (**H**) Alignment of deduced PHYB amino acid sequences of No-0, Kas-1, Edi-0, and Sha accessions in comparison to Col-0 showing conserved polymorphisms (red letters). (♯ indicates the laboratory accession of No-0). (**I-K**) Hypocotyl elongation of one-week old seedlings in No-0 related accessions at 17 (I), 22 (J) and 27ºC (K). (**L** and **M**) Upregulation of growth (L) and downregulation of defense responsive genes (M) in No-0-related ecotypes as measured by qRT-PCR (mean±SD of three biological replicates). (**N**-**P**) Natural accessions Kas-1, Edi-0, and Sha that show the similar amino acid sequence variations as No-0 show robust growth phenotypes (N) and enhanced disease susceptibility (O and P) phenocopying No-0.

In Figure A-C, E, F, and I-K, one-week-old seedlings grown at 22°C in SD were used for the experimental studies. *P≤0.05, **P≤0.01, ***P≤0.001 (Student's *t*-test) significantly different from either Col-0 (in I-M and P) or No-0 (in B, C, E and F).

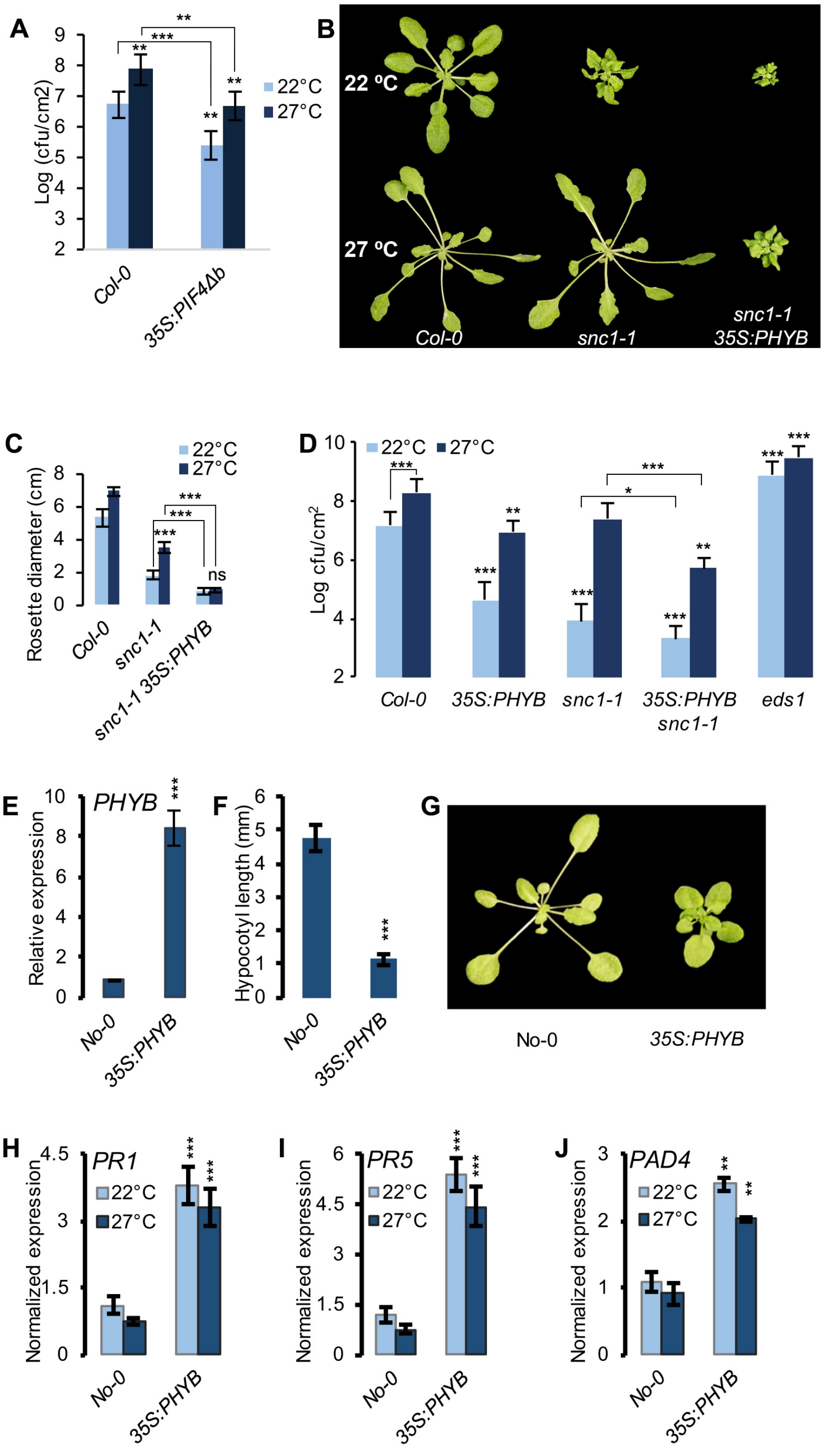

A
B
C
D
E
F
G
H
I
J
Log (cfu/cm2)
22°C
27°C
Col-0
35S:PIF4Δb
22 ºC
27 ºC
snc1-1
snc1-1 35S:PHYB
Rosette diameter (cm)
ns
Log cfu/cm2
35S:PHYB
eds1
PHYB
Relative expression
No-0
Hypocotyl length (mm)
PR1
PR5
PAD4
Normalized expression

Figure S4. *PHYB* overexpression leads to reduced growth and temperature resilient defense (Related to Figure 4)

(**A**) Overexpression of *PIF4Δb* (*35S:PIF4Δb*) leads to temperature resilient disease resistance to *Pto* DC3000 (A_{600}=0.002) (mean±SD; n=8). **P≤0.01, ***P≤0.001 (Student's *t*-test) significantly different from Col-0. (**B**) Rosette phenotype of four-weeks old plants shows *35S*:*PHYB* enhances *snc1-1* phenotype at 22°C and prevents suppression by elevated temperature (27°C). **(C**) Quantification of rosette diameter (mean±SD; n≥8) data showing *35S:PHYB* can completely prevent *snc1-1* phenotype suppression by elevated temperature. (**D**) *35S:PHYB* suppresses elevated temperature mediated defense response of *snc1-1* to *Pto* DC3000 (A_{600}=0.02; data is mean±SD; n≥8). Four-week-old plants grown at SD were used for the assay. *P≤0.05, **P≤0.01, ***P≤0.001 (Student's *t*-test) significantly different from either Col-0 or between indicated pairs. (**E**) Expression of *PHYB* in *35S*:*PHYB* transgenic line in No-0 background as measured by qRT-PCR (mean±SD of three biological replicates) from one-week-old seedlings grown at 22° C SD. (**F**) Hypocotyl data (mean±SD; n≥20) of one-week-old seedlings grown at 22° C in SD. (**G**) Rosette phenotype of three-week-old plants grown at 22°C SD. (**H-J**) Expression of defense responsive genes in as measured by qRT-PCR (mean±SD of three biological replicates) from one-week-old seedlings grown at 22° and 27°C. **p≤0.01 and ***P≤0.001 (Student's *t*-test) significantly different from No-0.

Supplemental Experimental Procedures

Plant material and growth conditions: All experiments unless otherwise specified, were performed on *Arabidopsis thaliana* laboratory accession Col-0. Mutants *pif4-101*[S1], *snc1-1*[S2] and *phyb-9*[S3], and transgenic lines *35S:PHYB-GFP*[S4] and *35S:PIF4-HA*[S5] were previously described. The natural accessions No-0 (N3081), Kas-1(N22638), Edi-0 (N22657), and Sha (N22652) were obtained from The European Arabidopsis Stock Centre (Nottingham). The transgenic line *35S:PHYB*$^{Col\text{-}0}$ in No-0 (ABO) overexpressing *PHYB* has been described[S6]. For all the experiments, seeds were stratified for 3-days at 4 °C in the dark and germinated at 22 ºC short photoperiod (SD; 8h light/16 h dark photoperiod). Later they were either retained at 22 °C SD or transferred to 17 ºC or 27°C SD depending on the experiment.

Hypocotyl measurement: For hypocotyl measurement, seeds were surface sterilized and germinated at 22 ºC SD on ½ MS media. Two days post germination, seedlings were shifted to 17 ºC, 22 °C or 27 °C SD for seven days before they were aligned on 1% agar plate and imaged using stereomicroscope. At least 20 seedlings were used to measure hypocotyl length using NIH ImageJ software (http://rsbweb.nih.gov/ij/).

Generation of double mutants: For the generation of *snc1-1 pif4-101* double mutant, *snc1-1* was crossed to *pif4-101.* Genotyping for *pif4-101* (primers 85, 213 and 214) and *snc1-1* (primers 124 and 125) mutations to identify homozygous double mutants. In case of *scn1-1 PHYBOE*, *snc1-1* was crossed to *35S:PHYB-GFP* to get F1, and resulting F2 seeds were screened at 27 °C and seedlings with short hypocotyl were transferred to soil. Later, in adult stage these plants were genotyped for *snc1-1* mutation (primers 124 and 125), and in the next generation several plants with *snc1-1* mutation were screened on MS-kanamycin to get plants homozygous at the *35S:PHYB-GFP locus.*

Vector construction and generation of transgenic lines: Full length *PIF4* cDNA was amplified using oligo nucleotide primers 298 and 299 and cloned into pENTR/D-TOPO (Invitrogen) to generate *pENTR-PIF4*. Oligo nucleotide primers 301 and 302 were used to delete the basic domain of *PIF4* in *pENTR-PIF4* to get *pENTR-PIF4Δb*. Both full length *PIF4* and *PIF4Δb* were used to generate binary vectors *35S:PIF4-Myc* and *35S:PIF4Δb-Myc* respectively through recombination with the binary destination vector pGWB417[S7] using LR clonase (Invitrogen). Oligos 117 and 118 were used to amplify P_{PIF4}*:PIF4* genomic fragment containing native promoter region to generate pENTR-P_{PIF4}*:PIF4.* Binary vector *pPIF4:PIF4-FLAG* was generated through recombination with the binary destination vector

pKGW3F. *PHYB* gene fragment was amplified using oligo nucleotide primers 292 and 293 and was cloned into pENTR/D-TOPO to generate *pENTR-PHYB,* which was used to generate *35S:PHYB-FLAG* construct through recombination into pK35GW3F. Oligo nucleotide primers 896 and 293 were used to amplify P_{PHYB}*:PHYB* fragments before cloning into pENTR/D-TOPO to generate *pENTR*- P_{PHYB}*:PHYB*, which was recombined into pGWB610[S8] to generate the binary vector pP_{PHYB}*:PHYB*. All recombinant clones were validated through restriction analysis and were sequence confirmed for the junction and reading frame. The binary vector clones were transformed into *Agrobacterium* GV3101, and transformed into either Col-0 or No-0 using floral dip method. Transgenics were selecteed on either MS+Kanamycin (50 µg/ml; for *35S:PIF4Δb*, P_{PIF4}*:PIF4-FLAG* and *35S:PHYB-FLAG* lines) or MS+Basta (10 µg/ml; for P_{PHYB}*:PHYB*) to identify transgenics. In T1 generation, individual transgenic plants were screened for single copy transgene insertion using KASP marker (iDna Genetics). In the next generation, several plants from single copy transgenic lines were assayed to identify homozygous lines.

Pathogen assays: Bacterial infection assays using *Pseudomonas syringae pv. tomato* DC3000 (Pst DC3000) strain were done by spray inoculation of 4 week old plants either grown continuously at 22 ºC or shifted from 22 ºC to 27 ºC for three days before infection. Bacteria were adjusted to OD600 = 0.02 in 10 mM $MgCl_2$ with 0.04% Silwet L-77. Three days post inoculation, leaf discs from three leaves were collected, and bacteria was extracted by shaking the leaf dics in 10mM $MgCl_2$ with 0.01% Silwett L-77 at 28°C for 1 h. Bacterial titre was determined by plating a serial dilution of the suspension onto NYGA plates with Rifampicin (50µg/ml) and incubated for two days at 28°C. In all the experiments *eds1* was used as a positive control for susceptibility. Significant differences to the corresponding wild type were analyzed using Student's *t*-test or 2-way ANOVA analysis with Tukey's multiple comparison test as specified in the legends.

RNA expression analysis: For gene expression analysis using quantitative-PCR, RNA was extracted using RNeasy Plant mini kit (Qiagen) with on-column DNase I digestion according to the manufacturer's instructions). RNA was quantified using NanoDrop, and approximately 1.5 µg of total RNA was converted into cDNA using Superscript III reverse transcriptase (Invitrogen) and oligo *dT* according to the manufacturer instructions. cDNA was diluted 1:20 and 2.0µl was used for qPCR using 2X SYBR Greem Master Mix kit in Roche Lightcycler 480. Quantitative RT-PCR experiments were performed in Light Cycler LC480 using Light

Cycler 480 SYBR Green I Master (Roche). *Ef1α* (AT5G60390) was used as internal control for normalization. Details of the oligo nucleotide primers used are provided below. Primers were designed using the Primer3-BLAST (http://www.ncbi.nlm.nih.gov/tools/primer-blast/).

No-0 × Col-0 F2 co-segregation analysis: To determine the association of growth and disease resistance traits to the *PHYB* allelic variants, F2 seeds were sown onto soil for 10 days at 22 ºC SD, and at least 100 seedlings with short and long hypocotyls were identified and transferred to separate trays, and grown for a further three-weeks. These plants were assayed for resistance to *Pst* DC3000 along with parental controls (No-0 and Col-0) and *eds1* as positive control for susceptibility. All the plants were SNP genotyped for *PHYB* allele using KASP marker assay. Seedlings with long hypocotyls were co-segregated with $PHYB^{No\text{-}0}$ and disease susceptibility, whereas seedlings with short hypocotyls were co-segregated with $PHYB^{Col\text{-}0}$ and disease resistance.

RNAseq analysis: For RNA sequencing analysis total RNA was extracted from 10-day-old seedlings grown on ½ MS solid medium using the RNeasy Plant Mini Kit (Qiagen). Sequencing was performed at The Genome Analysis Centre using Illumina HiSeq 2500 using 50 bp single-end sequencing. TopHat v2.0.8 was used to align the reads to the Arabidopsis reference genome (TAIR 10). A differential expression analysis was run using Cuffdiff v2.0.2. Significance of expression change was determined based on the p-value corrected for multiple hypothesis testing and a false discovery rate of 0.05. Differential gene expression analysis was done on the RPKM values generated. Strand NGS was used to analyze differential gene expression and further detailed analysis of the RNA-seq data.

List of oligonucleotides used in this study

Target	Oligo.	Oligo Sequence (5'…………..3')	Purpose
P_{PIF4}:PIF4-F	117	*CACCGCCTGTTTAATTGGTGCTTGGTCAATTACG*	Native expression
P_{PIF4}:PIF4-R	118	*GTGGTCCAAACGAGAACCGTCGGTGGTCT*	Native expression
PHYB-F	292	*CACCAAACGGCATGGTTTCC*	Overexpression
PHYB-R	293	*ATATGGCATCATCAGCATCATGT*	Overexpression
PIF4-F	298	*CACCTTTCTGTCTGTACCCAAA*	Overexpression
PIF4-R	299	*GTGGTCCAAACGAGAACCGTCGG*	Overexpression
PIF4-Δb	301	GCAGCTGAAGTTGATAGGATCAATGAGAGAATG	Overexpression
PIF4-Δb	302	*ATTGATCCTATCAACTTCAGCTGCTCGACTCCTTC*	Overexpression
PHYB-F	896	*CACCCGAATAAGAGAAGGAATTAC*	Complementation
PHYB-R	293	*ATATGGCATCATCAGCATCATGT*	Complementation
PIF4-LP	213	*AATACATTTTGCAGGCAATCG*	Genotyping *pif4-101*
PIF4-RP	214	*CGTAATGAAGTTGCACGTTTACTC*	Genotyping *pif4-101*
PIF4-LB	85	*TTCATAACCAATCTCGATACAC*	Genotyping *pif4-101*
SNC1-FP	124	*ATACGTTTGCCATTCGAGGA*	Genotyping *snc1-1*
SNC1-RP	125	*ACCAGAGTTCCTTCCCACAG*	Genotyping *snc1-1*
PHYB-F	825	*CATTTGGCAATGCAGGGGAC*	Q-PCR
PHYB-R	826	*GTCCAACAAAACAAACGCCG*	Q-PCR
PIF4-F	766	*ACCTCAGAGACGGTTAAGCC*	Q-PCR
PIF4-R	767	*TGGAGGAGGCATGACTTGAG*	Q-PCR
ATHB2-F	667	*CCGTCGGCTACAAAAAGAAG*	Q-PCR
ATHB2-R	668	*GAAGGGCACATGGTCAAAGT*	Q-PCR
EXP8-F	633	*CTCTTTCCGAAGAGTACCATGT*	Q-PCR
EXP8-R	634	*GTGTACGTCTCCTGCTCCTC*	Q-PCR
XTR7-F	1127	*CGGCTTGCACAGCCTCTT*	Q-PCR
XTR7-R	1128	*TCGGTTGCCACTTGCAATT*	Q-PCR
PR1-F	18	*ACCAGGCACGAGGAGCGGTA*	Q-PCR
PR1-R	19	*TCCCCGTAAGGCCCACCAGA*	Q-PCR
PR5-F	20	*ACCCACAGCACAGAGACACACA*	Q-PCR
PR5-R	21	*TGGCCATAACAGCAATGCCGC*	Q-PCR
EDS1-F	140	*GAAGGGATGCTTGGAGAATG*	Q-PCR
EDS1-R	141	*AGTCTCGCAGAGGAGAATGC*	Q-PCR
PAD4-F	315	*TGGTGACGAAGAAGGAGGTT*	Q-PCR
PAD4-R	316	*TCCATTGCGTCACTCTCATC*	Q-PCR
PBS3-F	323	*TGAGTCAAGCGAAGCTCGTA*	Q-PCR
PBS3-R	324	*ATCGATCCGTCTTTGAATCG*	Q-PCR
SID2-F	351	*TCTCAATTGGCAGGGAGACT*	Q-PCR
SID2-R	352	*AAGCCTTGCTTCTTCTGCTG*	Q-PCR

Supplemental references

S1. Lorrain, S., Allen, T., Duek, P.D., Whitelam, G.C., and Fankhauser, C. (2008). Phytochrome-mediated inhibition of shade avoidance involves degradation of growth-promoting bHLH transcription factors. Plant J. *53*, 312–23.

S2. Zhang. Y., Goritschnig. S., Dong. X., and Li, X. (2003). A Gain-of-function mutation in a plant disease resistance gene leads to constitutive activation of downstream signal transduction pathways in suppressor of npr1-1, constitutive 1. Plant Cell *15*, 2636–46.

S3. Reed, J.W., Nagpal, P., Poole, D.S., Furuya, M., and Chory, J. (1993). Mutations in the gene for the red/far-red light receptor phytochrome B alter cell elongation and physiological responses throughout Arabidopsis development. Plant Cell *5*, 147–57.

S4. Ádám, É., Kircher, S., Liu, P., Mérai, Z., González-Schain, N., Hörner, M., Viczián, A., Monte, E., Sharrock, R.A., Schäfer, E., et al. (2013). Comparative functional analysis of full-length and N-terminal fragments of phytochrome C, D and E in red light-induced signaling. New Phytol. *200*, 86–96.

S5. Nozue, K., Covington, M.F., Duek, P.D., Lorrain, S., Fankhauser, C., Harmer, S.L., and Maloof, J.N. (2007). Rhythmic growth explained by coincidence between internal and external cues. Nature *448*, 358–61.

S6. Wagner, D., Tepperman, J.M., and Quail, P.H. (1991). Overexpression of phytochrome B induces a short hypocotyl phenotype in transgenic Arabidopsis. Plant Cell *3*, 1275–88.

S7. Nakagawa, T., Suzuki, T., Murata, S., Nakamura, S., Hino, T., Maeo, K., Tabata, R., Kawai, T., Tanaka, K., Niwa, Y., et al. (2007). Improved gateway binary vectors: high-performance vectors for creation of fusion constructs in transgenic analysis of plants. Biosci. Biotechnol. Biochem. *71*, 2095–100.

S8. Nakamura, S., Mano, S., Tanaka, Y., Ohnishi, M., Nakamori, C., Araki, M., Niwa, T., Nishimura, M., Kaminaka, H., Nakagawa, T., et al. (2014). Gateway binary vectors with the bialaphos resistance gene, bar, as a selection marker for plant transformation. Biosci. Biotechnol. Biochem. *74*, 1315–9.
