# Supplementary material for: PIF4 Coordinates Thermosensory Growth and Immunity in Arabidopsis
Source: Curr Biol. 2017 Jan 23;27(2):243–9. doi: 10.1016/j.cub.2016.11.012 (PMC5266789; doi:10.1016/j.cub.2016.11.012)
Supplement: Document S2. Article plus Supplemental Information [file mmc4.pdf]

Report

Current Biology

PIF4 Coordinates Thermosensory Growth and Immunity in *Arabidopsis*

Graphical Abstract

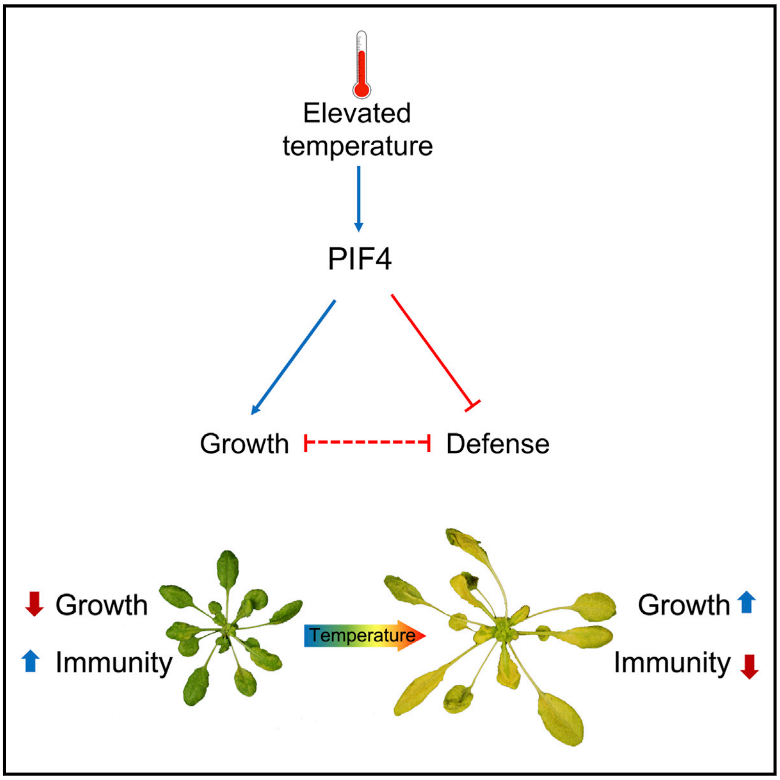

Authors

Sreeramaiah N. Gangappa, Souha Berriri, S. Vinod Kumar

Correspondence

vinod.kumar@jic.ac.uk

In Brief

Gangappa et al. show that the transcription factor PIF4 coordinates thermosensory growth and immunity. PIF4 acts as a negative regulator of plant immunity, and modulation of its function alters the balance between growth and defense. Importantly, natural variation of PIF4 signaling underlies growth-defense balance in *Arabidopsis* natural strains.

Highlights

- Transcription factor PIF4 coordinates thermosensory growth and immunity
- PIF4 negatively regulates immunity, while promoting growth
- Natural variation of PIF4 signaling underlies growth-defense balance in the wild
- Modulation of PIF4 signaling alters temperature sensitivity of disease resistance

Gangappa et al., 2017, Current Biology *27*, 243–249
January 23, 2017 © 2016 The Author(s). Published by Elsevier Ltd.
http://dx.doi.org/10.1016/j.cub.2016.11.012

Current Biology
Report

PIF4 Coordinates Thermosensory Growth and Immunity in *Arabidopsis*

Sreeramaiah N. Gangappa,[1] Souha Berriri,[1] and S. Vinod Kumar[1,2,*]
[1]Cell and Developmental Biology Department, John Innes Centre, Norwich NR4 7UH, UK
[2]Lead Contact
*Correspondence: vinod.kumar@jic.ac.uk
http://dx.doi.org/10.1016/j.cub.2016.11.012

SUMMARY

Temperature is a key seasonal signal that shapes plant growth. Elevated ambient temperature accelerates growth and developmental transitions [1] while compromising plant defenses, leading to increased susceptibility [2, 3]. Suppression of immunity at elevated temperature is at the interface of trade-off between growth and defense [2, 4]. Climate change and the increase in average growth-season temperatures threaten biodiversity and food security [5, 6]. Despite its significance, the molecular mechanisms that link thermosensory growth and defense responses are not known. Here we show that PHYTOCHROME INTERACTING FACTOR 4 (PIF4)-mediated thermosensory growth and architecture adaptations are directly linked to suppression of immunity at elevated temperature. PIF4 positively regulates growth and development and negatively regulates immunity. We also show that natural variation of PIF4-mediated temperature response underlies variation in the balance between growth and defense among *Arabidopsis* natural strains. Importantly, we find that modulation of PIF4 function alters temperature sensitivity of defense. Perturbation of PIF4-mediated growth has resulted in temperature-resilient disease resistance. This study reveals a molecular link between thermosensory growth and immunity in plants. Elucidation of the molecular mechanisms that define environmental signal integration is key to the development of novel strategies for breeding temperature-resilient disease resistance in crops.

RESULTS AND DISCUSSION

PIF4 Signaling Is Required for Temperature-Induced Suppression of the *snc1-1* Phenotype

Elevated temperature in spring promotes growth and accelerates developmental transitions [1], whereas it strongly suppresses defense responses. Trade-off between growth and immunity underlies the compromised resistance at higher temperatures [2]. One of the well-studied examples of temperature modulation of immunity is the suppression of resistance mediated by nucleotide-binding and leucine-rich repeat (NB-LRR) proteins such as SNC1 (SUPPRESSOR OF npr1-1, CONSTITUTIVE 1). The *snc1-1* mutation leads to constitutive activation of defense responses and severe growth defects [7], both of which are completely suppressed at higher ambient temperature [8]. The molecular mechanisms underlying immunity suppression by elevated temperature are not well understood. PHYTOCHROME INTERACTING FACTOR 4 (PIF4), a basic-helix-loop-helix (bHLH) transcription factor, controls thermosensory growth and architecture adaptations as well as reproductive transition in *Arabidopsis* [9, 10] and functions as an integrator of environmental cues [11, 12]. To test whether PIF4-mediated thermosensory signaling is involved in the modulation of immunity at elevated temperature, we studied the suppression of SNC1-mediated defense responses in the *snc1-1 pif4-101* double mutant. Growth defects of *snc1-1*, not the *snc1-1 pif4-101* double mutant, were suppressed by growth at 27°C (Figure 1A; Figures S1A–S1C). Increased resistance to *Pseudomonas syringae* pv. tomato (*Pto*) DC3000 of *snc1-1*, not *snc1-1 pif4-101*, is suppressed to wild-type levels at 27°C (Figure 1B). Further, gene expression analyses by qRT-PCR analysis of *PR1* and *PR5* (Figures 1C and 1D) have confirmed that the temperature-induced suppression of constitutively expressed defense genes in *snc1-1* was also *PIF4* dependent. Taken together, these results show that PIF4-mediated thermosensory signaling plays an important role in the suppression of defense by elevated temperature.

PIF4 Is a Negative Regulator of Immunity

The above results led us to hypothesize that PIF4 signaling could modulate defense responses. Gene expression analysis by qRT-PCR on 7-day-old seedlings grown at 22°C showed that whereas the expression of PIF4 target genes related to growth such as *ATHB2*, *EXP8*, and *XTR7* were downregulated in the *pif4-101* mutant as expected (Figure 2A), defense-related genes such as *PR1*, *PR5*, and *PBS3* were upregulated (Figure 2B), showing that PIF4 modulates immunity in *Arabidopsis*. Furthermore, RNA-sequencing (RNA-seq) analysis showed that genes that are upregulated in *pif4-101* were significantly enriched for defense-related Gene Ontology (GO) terms (Figure 2C; Data S1). Accordingly, the *pif4-101* and *pifQ* (*pif1 pif3 pif4 pif5*) quadruple mutant [13] showed increased resistance to *Pto* DC3000 when challenged with a lower inoculum (A_{600} 0.002) (Figure 2D) but not significantly when a higher bacterial titer (A_{600} 0.02) was used (Figure S1D). Together, the modulation of defense gene expression and alteration of disease resistance in the mutants show that PIF4 acts as a negative regulator of immunity.

This is an open access article under the CC BY license (http://creativecommons.org/licenses/by/4.0/).

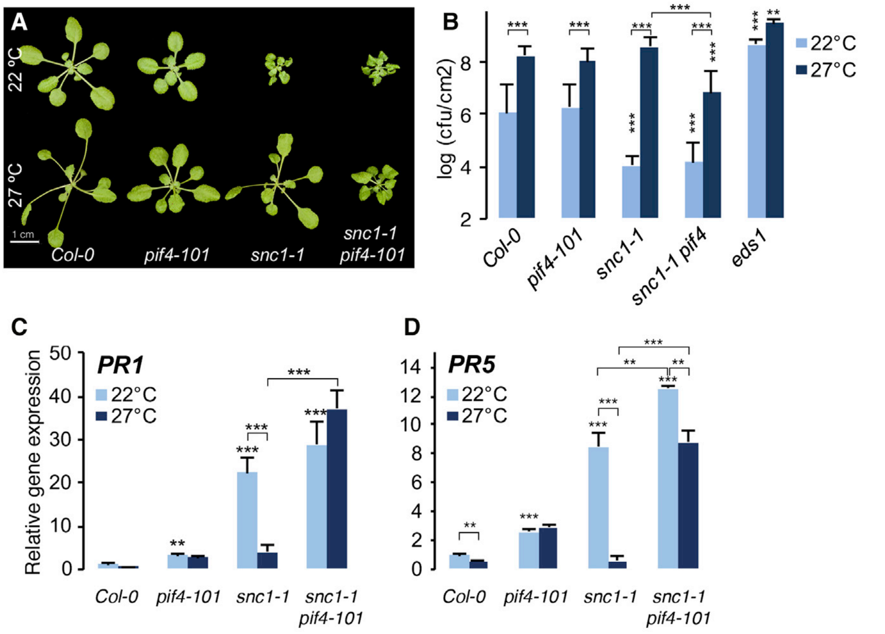

Figure 1. PIF4 Is Essential for the Suppression of Immunity by Elevated Temperature

Analysis of *snc1-1 pif4-101* double mutants shows that temperature-induced suppression of *snc1-1* phenotypes is PIF4 dependent.

(A) Morphological phenotypes of 4-week-old plants grown at 22°C and 27°C under a short-day photoperiod.

(B) Temperature-induced suppression of disease resistance of *snc1-1* is PIF4 dependent. Resistance phenotype of the indicated genotypes to *P. syringae pv.* tomato (*Pto*) DC3000 (A_{600} 0.02) at 22°C and 27°C (mean ± SD; n ≥ 8). **p ≤ 0.01, ***p ≤ 0.001 (two-way ANOVA with Tukey's multiple comparison test) compared to the corresponding Col-0 or as indicated; cfu, colony forming unit.

(C and D) Gene expression analysis of defense marker genes *PR1* (C) and *PR5* (D) by qRT-PCR (mean ± SD of three biological replicates) from 3-week-old plants. **p ≤ 0.01, ***p ≤ 0.001 (two-way ANOVA with Tukey's multiple comparison test) significantly different from either Col-0 or between the indicated pairs.

See also Figure S1.

PIF4, a bHLH transcription factor [14], functions cooperatively with other PIFs [11, 15] as well as with other proteins involved in growth and immunity [12, 16, 17]. The modest increase in resistance in *pif4* and *pifQ* could be reflecting the quantitative contribution of these, including PIF7 [14], to defense modulation. The bHLH transcription factors function as hetero- or homo-dimers and require the basic (b) domain for DNA binding [14]. Dimerization with a protein lacking the basic domain renders them non-DNA binding and therefore non-functional [18, 19]. Therefore, with the aim of producing a dominant negative with little or no PIF function, we generated a PIF4 variant lacking the basic domain, hereafter referred to as PIF4Δb (Figure 2E). When overexpressed, *PIF4Δb* resulted in strong suppression of growth (Figure 2F; Figures S1E and S1F), suggesting that PIF4Δb acts as a dominant negative as expected. Further substantiating this, PIF4Δb strongly suppressed the enhanced growth promoted by 35S:*PIF4-HA* (hemagglutinin) (Figure S1G). Consistent with this, *35S:PIF4Δb* led to downregulation of growth-related genes and upregulation of defense genes (Figure 2G; Figures S1H and S1I) and enhanced resistance to *Pto* DC3000 (Figure 2H), further substantiating the role of PIF4 in modulating immunity. In a complementary experiment, we analyzed a P_{PIF4}:*PIF4-FLAG* transgenic line showing *PIF4* overexpression (*PIF4-OE*) (Figure S1J) that showed enhanced elongation growth (Figure 2I; Figure S1K). Interestingly, *PIF4-OE* showed increased expression of growth-related genes and reduced defense gene expression (Figures S1L and S1M) as well as increased susceptibility to *Pto* DC3000 (Figure 2J), showing that PIF4 is sufficient to modulate immunity.

The photoreceptor phytochrome B (PHYB) regulates PIF transcription factors at the protein level through promoting light-dependent protein degradation. Loss-of-function *phyb* mutants show exaggerated PIF-mediated growth [11, 20, 21]. Supporting our above results and consistent with earlier reports [22], *phyb*-9 showed increased susceptibility to *Pto* DC3000 (Figure 2K). Conversely, *35S:PHYB-FLAG* transgenic lines (Figure S1N) showed reduced growth (Figure S1O) concomitant with decreased expression of growth-related genes (Figure S1P). In line with the role of the PIF-PHYB module in growth-defense balance, the *35S:PHYB-FLAG* lines showed increased defense gene expression (Figures S1Q and S1R) and enhanced resistance to *Pto* DC3000 (Figure 2K). These results further established the role of PIF4 signaling in coordinating plant growth and immunity.

Natural Variation in PIF4 Signaling Underlies Growth-Defense Balance

In nature, growth and development are fine-tuned to suit the prevailing local environmental conditions [23, 24]. We examined natural variation of thermosensory growth in *Arabidopsis* in relation to defense. The natural accession Nossen (*No-0*) showed robust growth (Figure 3A), enhanced thermosensory flowering (Figure 3B), and enhanced temperature-induced hypocotyl elongation (Figure 3C) under a short-day photoperiod, phenocopying Columbia (*Col*)*-0* plants grown at warmer temperatures. Consistent with this, expression of growth-related genes was significantly upregulated in *No-0* (Figure 3D). This was accompanied by reduced expression of key defense marker genes (Figure 3E). Further, whole-genome transcriptome analysis by RNA-seq showed that the genes downregulated in *No-0* were enriched for defense-related GO terms (Figure 3F; Data S2). Accordingly, we found that *No-0* is more susceptible to *Pto* DC3000 (Figure 3G), suggesting that enhanced thermosensory growth in *No-0* leads to compromised basal immunity.

Growth and defense phenotypes of *No-0* were reminiscent of the *phyb*-9 mutant (Figures S2A–S2C). Moreover, F_1 seedlings from a *No-0* × *phyb*-9 cross did not show complementation of the *phyb-9* phenotype (Figure S2D), suggesting that PHYB function could be compromised in *No-0*. *PHYB* expression in *No-0*, however, was comparable to *Col-0* (Figure S2E). To test whether

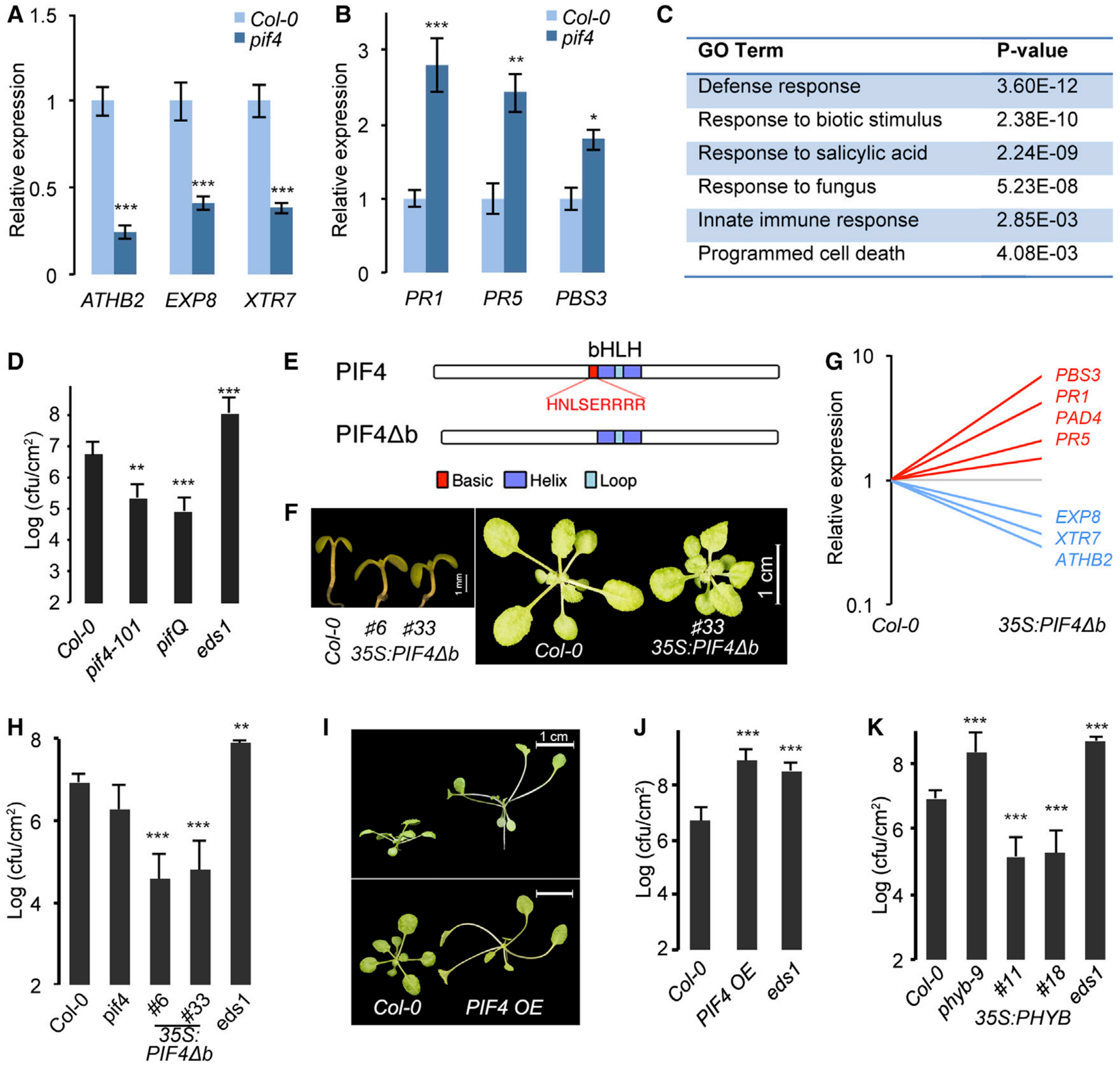

Figure 2. PIF4 Is a Negative Regulator of Immunity

(A and B) Downregulation of growth-related genes (A) and upregulation of defense genes (B) in *pif4-101* as shown by qRT-PCR (mean ± SD of three biological replicates) from 1-week-old seedlings grown at 22°C under a short-day photoperiod.

(C) Enrichment of defense GO terms in genes upregulated in 1-week-old *pif4-101* (Dataset S1) seedlings grown at 22°C under a short-day photoperiod.

(D) Increased disease resistance of *pif4-101* and *pifQ* mutants to *Pto* DC3000 (A_{600} 0.002; mean ± SD; n = 8).

(E) Schematic representation of PIF4Δb, which lacks the basic domain.

(F) Reduced hypocotyl elongation growth and rosette phenotype in two independent lines overexpressing *PIF4Δb*.

(G) Overexpression of *PIF4Δb* leads to downregulation of growth (blue) and upregulation of defense (red) genes (data are the average of three biological replicates; see also Figure S1) in 22°C short-day-grown seedlings for 1 week.

(H) Disease-resistance phenotype of 35S:*PIF4Δb* to *Pto* DC3000 (A_{600} 0.02; mean ± SD; n $\geq$ 12).

(I) *PIF4-FLAG* OE showing enhanced elongation growth.

(J and K) Disease-resistance phenotype of *PIF4-FLAG* OE (J) and *35S:PHYB-FLAG* (K) lines to *Pto* DC3000 (A_{600} 0.02; mean ± SD; n $\geq$ 12).

*p $\leq$ 0.05, **p $\leq$ 0.01, ***p $\leq$ 0.001 (Student's t test) significantly different from Col-0. In (D), (H), (J), and (K), plants grown at 22°C under a short-day photoperiod for 4 weeks were used for the resistance assays. See also Figure S1.

the altered PHYB function is due to variation at the *PHYB* locus, we carried out a comparative sequence analysis. We found that the *PHYB* locus of *No-0*, hereafter referred to as *PHYB*$^{No\text{-}0}$, is polymorphic. *PHYB*$^{No\text{-}0}$ has a 15 bp deletion, causing an in-frame deletion (ΔSGGGR) at the N terminus, and two non-synonymous SNPs leading to amino acid substitutions I143L and L1072V (Figure 3H), which were previously shown to be associated with PHYB function [25]. Interestingly, hierarchical clustering of *Arabidopsis* natural accessions and mutants for light responses resulted in No-0 and *phyb* alleles, particularly *phyb-9*, to cluster together [26], providing further evidence that No-0 is perturbed in PHYB function.

To test whether the variant *PHYB*$^{No\text{-}0}$ allele underlies the variation in growth and immunity, we analyzed the F_2 population of a

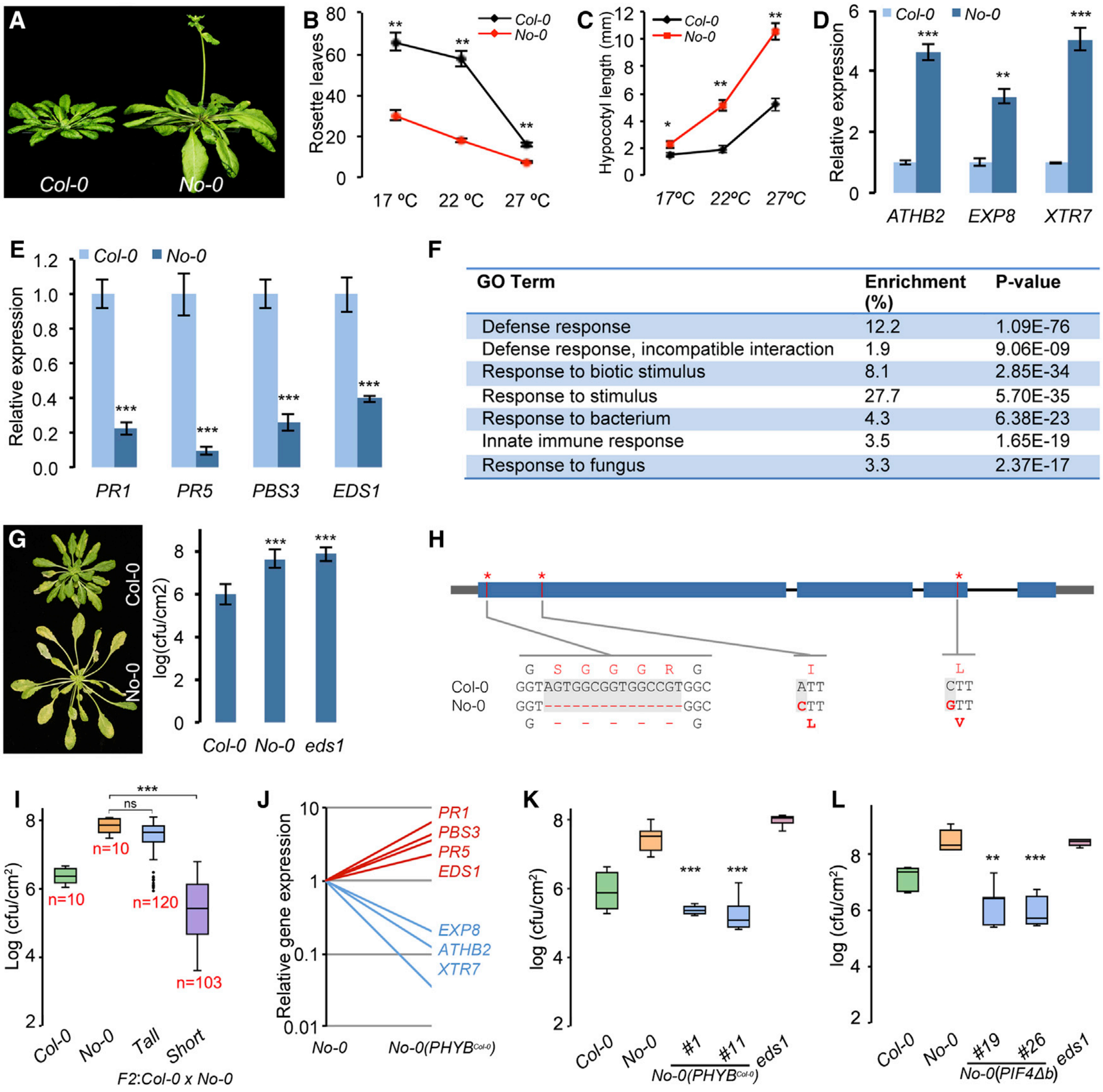

Figure 3. Natural Variation of PIF4-Mediated Thermosensory Growth and Immunity

(A–C) No-0 shows robust growth (A) and enhanced thermosensory flowering (B) and hypocotyl growth (C).

(D and E) Gene expression of growth (D) and defense markers (E) in No-0 as shown by qRT-PCR (mean ± SD of three biological replicates).

(F) GO analysis showing that genes downregulated in No-0 (see also Data S2) are enriched for defense GO terms.

(G) No-0 shows increased susceptibility to *Pto* DC3000 (A_{600} 0.02; mean ± SD; n $\geq$ 10).

(H) Diagrammatic representation of $PHYB^{No\text{-}0}$ showing polymorphisms at the nucleotide and amino acid level.

(I) Resistance to *Pto* DC3000 (A_{600} 0.02) of F_2 segregants of a Col-0 × No-0 cross showing co-segregation of growth and defense phenotypes. Three-week-old plants grown in 22°C under a short-day photoperiod were used for the experiment.

(J) Transgenic expression of $PHYB{:}PHYB^{Col\text{-}0}$ fully complements gene expression phenotypes of No-0 (mean of three biological replicates; see also Figure S2).

(K) Disease-resistance phenotypes of two independent transgenic lines of No-0 complemented with $PHYB{:}PHYB^{Col\text{-}0}$.

(L) Overexpression of *PIF4Δb* in No-0 leads to increased resistance to *Pto* DC3000 (A_{600} 0.02) (two independent transgenic lines are shown).

In (C)–(E) and (J), 1-week-old seedlings grown at 22°C under a short-day photoperiod were used for the experiments. In (G), (K), and (L), 4-week-old plants grown at 22°C under a short-day photoperiod were used for the resistance assays. *p $\leq$ 0.05, **p $\leq$ 0.01, ***p $\leq$ 0.001 (Student's t test) significantly different from either Col-0 (in B–E and G) or No-0 (in I, K, and L). See also Figures S2 and S3.

cross between *Col-0* and *No-0* for growth and defense phenotypes (Figure 3I). Long-hypocotyl and disease-susceptibility phenotypes were strongly associated with $PHYB^{No\text{-}0}$, whereas short hypocotyl and disease resistance were associated with $PHYB^{Col\text{-}0}$ (Figure 3I; Figure S2F), suggesting that PHYB is the major determinant of phenotypic variation. It is also possible that other factors such as *PIF4* itself could add to *PHYB* in balancing growth and defense in No-0. Further, P_{PHYB}:$PHYB^{Col\text{-}0}$ fully complemented the growth and gene expression phenotypes of *No-0* (Figure 3J; Figures S2G–S2J). Moreover, the complemented lines showed increased resistance to *Pto* DC3000, which was comparable to *Col-0* (Figure 3K). Together, these results confirmed that the hypomorphic $PHYB^{No\text{-}0}$ underlies the altered growth-defense balance.

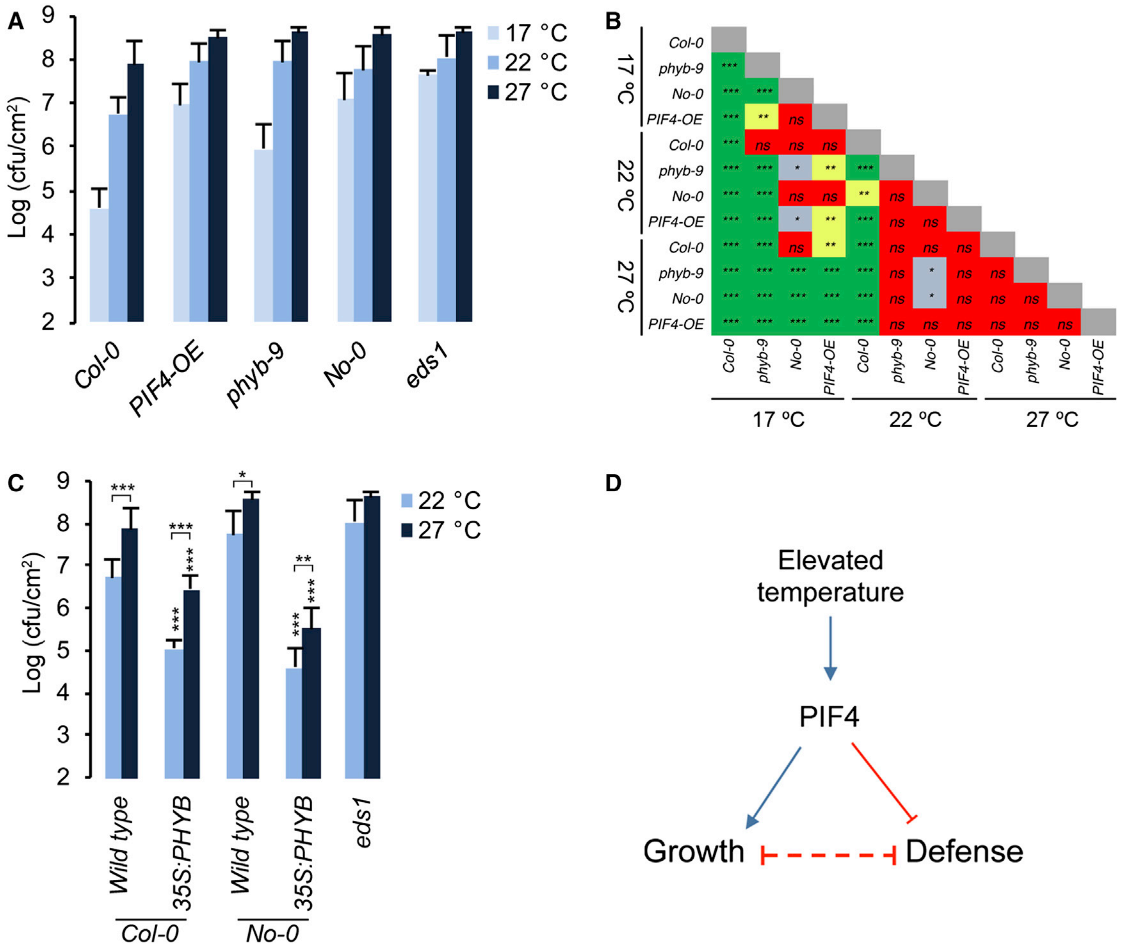

Figure 4. PIF4-Mediated Thermosensory Signaling Modulates Temperature Sensitivity of Immunity

(A) Increased PIF4 function (in *PIF4-OE*, *phyb-9*, and No-0) leads to increased susceptibility to *Pto* DC3000 (A_{600} 0.002) at lower temperatures, phenocopying wild-type plants grown at higher temperature.

(B) Two-way ANOVA analysis with Tukey's multiple comparison test of data from (A); *$p \leq 0.05$, **$p \leq 0.01$, ***$p \leq 0.001$; ns, not significant.

(C) *PHYB* overexpression leads to temperature-resilient disease resistance to *Pto* DC3000 (A_{600} 0.002) (mean ± SD; n = 8). *$p \leq 0.05$, **$p \leq 0.01$, ***$p \leq 0.001$ (two-way ANOVA with Tukey's multiple comparison test) significantly different from either Col-0 at respective temperatures or between the indicated pairs. See also Figure S4.

(D) Model showing PIF4 function at the interface of growth and defense responses. While promoting thermosensory growth, PIF4 negatively regulates immunity. See also Figure S4.

Because PHYB negatively regulates PIF4, we hypothesized that the phenotypes of *No-0* could be due to enhanced PIF4 function. Interestingly, *No-0* also showed increased expression of *PIF4* (Figure S3A). Moreover, overexpression of the dominant-negative PIF4Δb (Figure S3B) strongly suppressed the growth phenotypes (Figures S3C and S3D). PIF4Δb transgenic lines showed reduced expression of growth-related genes (Figure S3E), while enhancing defense gene expression (Figure S3F) and disease resistance (Figure 3L). We therefore conclude that the altered growth-defense balance in *No-0* is due to increased PIF4 function, as a result of reduced PHYB-mediated repression. It could also be at least in part due to increased *PIF4* expression (Figure S3A).

To test whether *PHYB* allelic variation is reflected in altered growth-immunity balance more widely in nature, we analyzed the worldwide set of 96 *Arabidopsis* natural accessions [27]. Comparative analysis of deduced PHYB amino acid sequences (Figure S3G) identified Edinburgh (*Edi*)-*0*, Kashmir (*Kas*)-*1*, and Shakdara (*Sha*) to have similar PHYB protein as *No-0*, including the I143L and L1072V substitutions (Figure S3H). All three accessions showed enhanced elongation of hypocotyl (Figures S3I–S3K) and robust growth (Figure S3N) phenocopying *No-0*, which was accompanied by upregulated expression of growth-related genes (Figure S3L) and downregulation of defense-related genes (Figure S3M). Most importantly, these accessions showed increased susceptibility to *Pto* DC3000 (Figures S3O and S3P), confirming that the PHY-PIF signaling module coordinates growth and defense in the wild.

Modulation of PIF4 Signaling Alters Temperature-Induced Modulation of Immunity

The above results have clearly shown that PIF4 coordinates growth and defense responses. To test whether PIF4 signaling

also controls temperature sensitivity of defense, we studied disease resistance at elevated temperature. We tested whether increased PIF4 signaling could lead to increased susceptibility at lower temperature. When grown at 17°C, wild-type Col-0 shows increased resistance to *Pto* DC3000 (Figures 4A and 4B). Further substantiating the role of PIF4 in modulating defense, *PIF4-OE* showed increased susceptibility at 17°C and 22°C, phenocopying Col-0 plants grown at 22°C and 27°C, respectively (Figures 4A and 4B), showing that PIF4 is sufficient for mediating temperature-induced susceptibility. In line with this, *phyb-9* and *No-0* showed strongly reduced resistance to *Pto* DC3000 at lower temperature, phenocopying growth at elevated temperatures (Figures 4A and 4B). Consistent with the established signaling hierarchy, *PIF4-OE* showed reduced temperature sensitivity of resistance compared to *phyb-9*. Further, the *35S:PIF4*Δ*b* line showed significantly increased resistance even at 27°C compared to Col-0 (Figure S4A). Conversely, *35S:PHYB-GFP* [28] strongly enhanced *snc1-1* phenotypes at 22°C and prevented its suppression at 27°C (Figures S4B–S4D). Accordingly, a transgenic line overexpressing *PHYB*$^{Col\text{-}0}$ in *No-0* [29] strongly suppressed growth (Figures S4E–S4G), and has resulted in enhanced defense gene expression even at 27°C (Figures S4H–S4J). Importantly, this has resulted in temperature-resilient resistance to *Pto* DC3000 at 27°C (Figure 4C). Together, our data clearly show that the PIF4-mediated thermosensory signaling module is both essential and sufficient to modulate temperature sensitivity of defense responses.

Conclusions

Collectively, our data show that PIF4, a central component of temperature responses, coordinates thermosensory growth and immunity (Figure 4D). Natural variation of PIF4-mediated growth defines the balance between growth and immunity in the wild. To grow robustly and to effectively fend off pathogens are extremely desirable traits. However, trade-offs between these processes lead to optimization of growth and defense in nature, exemplified by strategies where reduced growth leads to a fitness advantage of being well protected from pathogens [24]. Similarly, robust growth at the cost of reduced defense could be beneficial when pathogen load is low or under conditions that restrict growth. Lower temperature and insufficient resources such as nutrients and light quality due to competition or seasonal fluctuations are growth limiting. PHYB is a major regulator that limits growth in response to the environment. Therefore, accessions such as *No-0* could have a fitness advantage through robust growth and shorter life cycle that could help evade pathogens. Conversely, enhanced PHYB-mediated growth restraint may be advantageous under warmer environments, especially in the context of climate change [5, 6]. Being a central environmental signaling hub, PIF4 could therefore be involved in coordinating growth and defense in response to a number of environmental signals including light quality and during shade-avoidance responses. Understanding the mechanistic framework of environmental signal integration will be vital for breeding climate-resilient crops. This study unravels such a mechanism whereby growth and defense responses are coordinated in response to the environment.

SUPPLEMENTAL INFORMATION

Supplemental Information includes Supplemental Experimental Procedures, four figures, and two data files and can be found with this article online at http://dx.doi.org/10.1016/j.cub.2016.11.012.

AUTHOR CONTRIBUTIONS

S.N.G. designed and performed most of the experiments and analyzed data. S.B. contributed to the experiments and data analysis. S.V.K. designed and supervised the study and analyzed data. S.N.G. and S.V.K. wrote the paper.

ACKNOWLEDGMENTS

This work was supported by Biotechnology and Biological Sciences Research Council (BBSRC) grant BB/I019022/1 and Institute Strategic Programme grants BB/J004588/1 and BB/J004553/1. S.N.G. is supported by European Commission H2020 MSCA Fellowship 656995. We thank Robert Sablowski, Cyril Zipfel, Jeremy Murray, Kirsten Bomblies, Martin Howard, Lars Ostergaard, Doris Lucyshyn, and Scott Boden for critical reading of the manuscript. We thank Laura Hebberecht Lopez for help with bacterial resistance assays. We thank the members of the S.V.K. laboratory for helpful discussions.

Received: September 19, 2016
Revised: November 1, 2016
Accepted: November 4, 2016
Published: December 29, 2016

REFERENCES

1. Quint, M., Delker, C., Franklin, K.A., Wigge, P.A., Halliday, K.J., and van Zanten, M. (2016). Molecular and genetic control of plant thermomorphogenesis. Nat. Plants *2*, 15190.
2. Alcázar, R., and Parker, J.E. (2011). The impact of temperature on balancing immune responsiveness and growth in *Arabidopsis*. Trends Plant Sci. *16*, 666–675.
3. Hua, J. (2013). Modulation of plant immunity by light, circadian rhythm, and temperature. Curr. Opin. Plant Biol. *16*, 406–413.
4. Huot, B., Yao, J., Montgomery, B.L., and He, S.Y. (2014). Growth-defense tradeoffs in plants: a balancing act to optimize fitness. Mol. Plant *7*, 1267–1287.
5. Battisti, D.S., and Naylor, R.L. (2009). Historical warnings of future food insecurity with unprecedented seasonal heat. Science *323*, 240–244.
6. Gornall, J., Betts, R., Burke, E., Clark, R., Camp, J., Willett, K., and Wiltshire, A. (2010). Implications of climate change for agricultural productivity in the early twenty-first century. Philos. Trans. R. Soc. Lond. B Biol. Sci. *365*, 2973–2989.
7. Zhang, Y., Goritschnig, S., Dong, X., and Li, X. (2003). A gain-of-function mutation in a plant disease resistance gene leads to constitutive activation of downstream signal transduction pathways in suppressor of npr1-1, constitutive 1. Plant Cell *15*, 2636–2646.
8. Zhu, Y., Qian, W., and Hua, J. (2010). Temperature modulates plant defense responses through NB-LRR proteins. PLoS Pathog. *6*, e1000844.
9. Koini, M.A., Alvey, L., Allen, T., Tilley, C.A., Harberd, N.P., Whitelam, G.C., and Franklin, K.A. (2009). High temperature-mediated adaptations in plant architecture require the bHLH transcription factor PIF4. Curr. Biol. *19*, 408–413.
10. Kumar, S.V., Lucyshyn, D., Jaeger, K.E., Alós, E., Alvey, E., Harberd, N.P., and Wigge, P.A. (2012). Transcription factor PIF4 controls the thermosensory activation of flowering. Nature *484*, 242–245.
11. Leivar, P., and Quail, P.H. (2011). PIFs: pivotal components in a cellular signaling hub. Trends Plant Sci. *16*, 19–28.
12. Leivar, P., and Monte, E. (2014). PIFs: systems integrators in plant development. Plant Cell *26*, 56–78.

13. Leivar, P., Monte, E., Oka, Y., Liu, T., Carle, C., Castillon, A., Huq, E., and Quail, P.H. (2008). Multiple phytochrome-interacting bHLH transcription factors repress premature seedling photomorphogenesis in darkness. Curr. Biol. *18*, 1815–1823.
14. Toledo-Ortiz, G., Huq, E., and Quail, P.H. (2003). The *Arabidopsis* basic/helix-loop-helix transcription factor family. Plant Cell *15*, 1749–1770.
15. Pfeiffer, A., Shi, H., Tepperman, J.M., Zhang, Y., and Quail, P.H. (2014). Combinatorial complexity in a transcriptionally centered signaling hub in *Arabidopsis*. Mol. Plant *7*, 1598–1618.
16. Oh, E., Zhu, J.-Y., and Wang, Z.-Y. (2012). Interaction between BZR1 and PIF4 integrates brassinosteroid and environmental responses. Nat. Cell Biol. *14*, 802–809.
17. Lozano-Durán, R., Macho, A.P., Boutrot, F., Segonzac, C., Somssich, I.E., and Zipfel, C. (2013). The transcriptional regulator BZR1 mediates trade-off between plant innate immunity and growth. eLife *2*, e00983.
18. Hornitschek, P., Lorrain, S., Zoete, V., Michielin, O., and Fankhauser, C. (2009). Inhibition of the shade avoidance response by formation of non-DNA binding bHLH heterodimers. EMBO J. *28*, 3893–3902.
19. Ishii, R., Isogaya, K., Seto, A., Koinuma, D., Watanabe, Y., Arisaka, F., Yaguchi, S., Ikushima, H., Dohmae, N., Miyazono, K., et al. (2012). Structure of a dominant-negative helix-loop-helix transcriptional regulator suggests mechanisms of autoinhibition. EMBO J. *31*, 2541–2552.
20. Li, L., Ljung, K., Breton, G., Schmitz, R.J., Pruneda-Paz, J., Cowing-Zitron, C., Cole, B.J., Ivans, L.J., Pedmale, U.V., Jung, H.S., et al. (2012). Linking photoreceptor excitation to changes in plant architecture. Genes Dev. *26*, 785–790.
21. Chen, M., and Chory, J. (2011). Phytochrome signaling mechanisms and the control of plant development. Trends Cell Biol. *21*, 664–671.
22. de Wit, M., Spoel, S.H., Sanchez-Perez, G.F., Gommers, C.M.M., Pieterse, C.M.J., Voesenek, L.A.C.J., and Pierik, R. (2013). Perception of low red:far-red ratio compromises both salicylic acid- and jasmonic acid-dependent pathogen defences in *Arabidopsis*. Plant J. *75*, 90–103.
23. Fournier-Level, A., Korte, A., Cooper, M.D., Nordborg, M., Schmitt, J., and Wilczek, A.M. (2011). A map of local adaptation in *Arabidopsis thaliana*. Science *334*, 86–89.
24. Todesco, M., Balasubramanian, S., Hu, T.T., Traw, M.B., Horton, M., Epple, P., Kuhns, C., Sureshkumar, S., Schwartz, C., Lanz, C., et al. (2010). Natural allelic variation underlying a major fitness trade-off in *Arabidopsis thaliana*. Nature *465*, 632–636.
25. Filiault, D.L., Wessinger, C.A., Dinneny, J.R., Lutes, J., Borevitz, J.O., Weigel, D., Chory, J., and Maloof, J.N. (2008). Amino acid polymorphisms in *Arabidopsis* phytochrome B cause differential responses to light. Proc. Natl. Acad. Sci. USA *105*, 3157–3162.
26. Maloof, J.N., Borevitz, J.O., Dabi, T., Lutes, J., Nehring, R.B., Redfern, J.L., Trainer, G.T., Wilson, J.M., Asami, T., Berry, C.C., et al. (2001). Natural variation in light sensitivity of *Arabidopsis*. Nat. Genet. *29*, 441–446.
27. Nordborg, M., Hu, T.T., Ishino, Y., Jhaveri, J., Toomajian, C., Zheng, H., Bakker, E., Calabrese, P., Gladstone, J., Goyal, R., et al. (2005). The pattern of polymorphism in *Arabidopsis thaliana*. PLoS Biol. *3*, e196.
28. Ádám, É., Kircher, S., Liu, P., Mérai, Z., González-Schain, N., Hörner, M., Viczián, A., Monte, E., Sharrock, R.A., Schäfer, E., and Nagy, F. (2013). Comparative functional analysis of full-length and N-terminal fragments of phytochrome C, D and E in red light-induced signaling. New Phytol. *200*, 86–96.
29. Wagner, D., Tepperman, J.M., and Quail, P.H. (1991). Overexpression of phytochrome B induces a short hypocotyl phenotype in transgenic *Arabidopsis*. Plant Cell *3*, 1275–1288.

Current Biology, Volume *27*

Supplemental Information

PIF4 Coordinates Thermosensory

Growth and Immunity in *Arabidopsis*

Sreeramaiah N. Gangappa, Souha Berriri, and S. Vinod Kumar

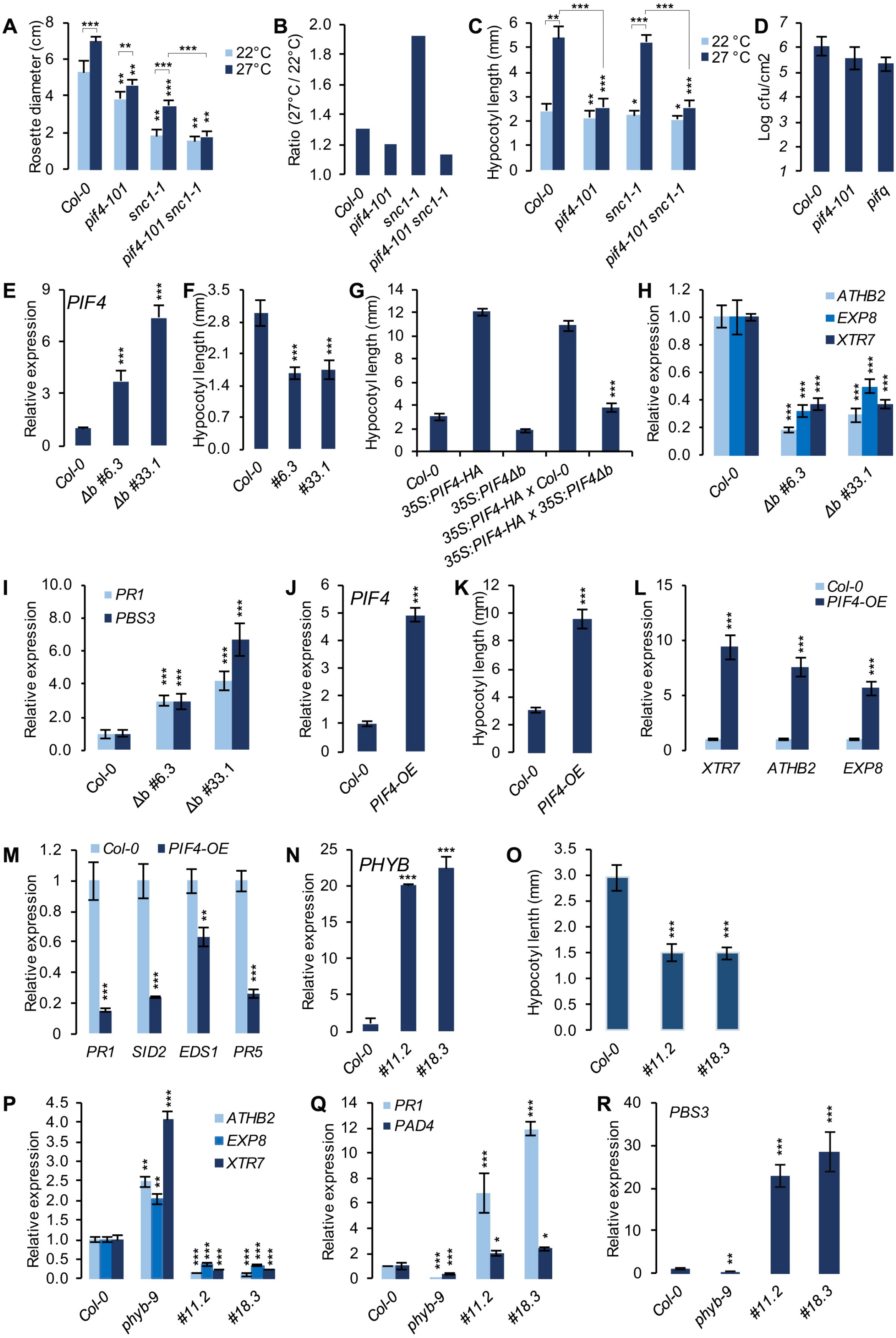

A
Rosette diameter (cm)
22°C
27°C
Col-0
pif4-101
snc1-1
pif4-101 snc1-1
B
Ratio (27°C / 22°C)
Col-0
pif4-101
snc1-1
pif4-101 snc1-1
C
Hypocotyl length (mm)
22 °C
27 °C
Col-0
pif4-101
snc1-1
pif4-101 snc1-1
D
Log cfu/cm2
Col-0
pif4-101
pifq
E
PIF4
Relative expression
Col-0
Δb #6.3
Δb #33.1
F
Hypocotyl length (mm)
Col-0
#6.3
#33.1
G
Hypocotyl length (mm)
Col-0
35S:PIF4-HA
35S:PIF4Δb
35S:PIF4-HA x Col-0
35S:PIF4-HA x 35S:PIF4Δb
H
Relative expression
ATHB2
EXP8
XTR7
Col-0
Δb #6.3
Δb #33.1
I
Relative expression
PR1
PBS3
Col-0
Δb #6.3
Δb #33.1
J
PIF4
Relative expression
Col-0
PIF4-OE
K
Hypocotyl length (mm)
Col-0
PIF4-OE
L
Relative expression
Col-0
PIF4-OE
XTR7
ATHB2
EXP8
M
Relative expression
Col-0
PIF4-OE
PR1
SID2
EDS1
PR5
N
PHYB
Relative expression
Col-0
#11.2
#18.3
O
Hypocotyl lenth (mm)
Col-0
#11.2
#18.3
P
Relative expression
ATHB2
EXP8
XTR7
Col-0
phyb-9
#11.2
#18.3
Q
Relative expression
PR1
PAD4
Col-0
phyb-9
#11.2
#18.3
R
PBS3
Relative expression
Col-0
phyb-9
#11.2
#18.3

Figure S1. PIF4 negatively regulates immunity (Related to Figure1 and Figure 2)

(**A-C**) PIF4 is essential for the elevated temperature mediated suppression of *snc1-1* growth phenotypes. Quantification of rosette diameter (A; mean±SD, n≥8), the response ratio (27°C/22°C; B), and hypocotyl elongation (**C;** mean±SD, n≥20). For the measurement of rosette diameter, plants grown at 22°C under short-day photoperiod for three-weeks were either retained at 22°C or exposed to 27°C for 6-days. On day 6 plants were photographed and diameter was measured using ImageJ software. (**D**) Resistance phenotype of *pif4-101* and *pifq* (*pif1 pif3 pif4 pif5*) mutants to *Pto* DC3000 (A_{600}=0.02; Mean±SD; n=6). Four-week-old plants grown at SD were used for the assay. (**E**) Expression of total *PIF4* (*PIF4* + *PIF4Δb*) in two independent *35S:PIF4Δb-Myc* (*35S:PIF4Δb*) lines (#6.3 and 33.1) as measured by qRT-PCR (mean±SD of three biological replicates). (**F**) Quantification of hypocotyl elongation (mean±SD; n≥20) in *35S:PIF4Δb* transgenic lines. (**G**) Overexpression of *PIF4Δb* strongly suppresses enhanced growth phenotypes of *35S:PIF4-HA.* Quantification of hypocotyl elongation (mean±SD; n≥20) of indicated genotypes. (**H** and **I**) Overexpression of *PIF4Δb-Myc* (35S:*PIF4Δb*) leads to downregulation of growth-related genes (**H**) and upregulation of defense responsive genes (**I**) as measured by qRT-PCR (mean±SD of three biological replicates). (**J**) Expression of *PIF4* in P_{PIF4}*:PIF4-FLAG* (*PIF4 OE*) transgenic line as measured by qRT-PCR (mean±SD of three biological replicates). (**K**) Quantification of hypocotyl elongation data (mean±SD; n≥20). (**L** and **M**) Increased expression of *PIF4* leads to upregulation of growth-related genes (**L**) and downregulation of defense (**M**) responsive genes (mean±SD of three biological replicates). (**N**) Expression of *PHYB* in two independent *35S:PHYB-FLAG (35S:PHYB)* transgenic lines (#11.2 and 18.3) as measured by qRT-PCR (mean±SD of three biological replicates). (**O**) Hypocotyl data (mean±SD; n≥20) of *35S:PHYB* seedlings. (**P-R**) Expression of growth (P) and defense responsive genes (Q and R) in *35S:PHYB* lines as measured by qRT-PCR (mean±SD of three biological replicates).

In Figure C and E-R, one-week-old seedlings grown at 22°C SD were used for the experiments. *P≤0.05, **P≤0.01, ***P≤0.001 (Student's *t*-test) significantly different from Col-0.

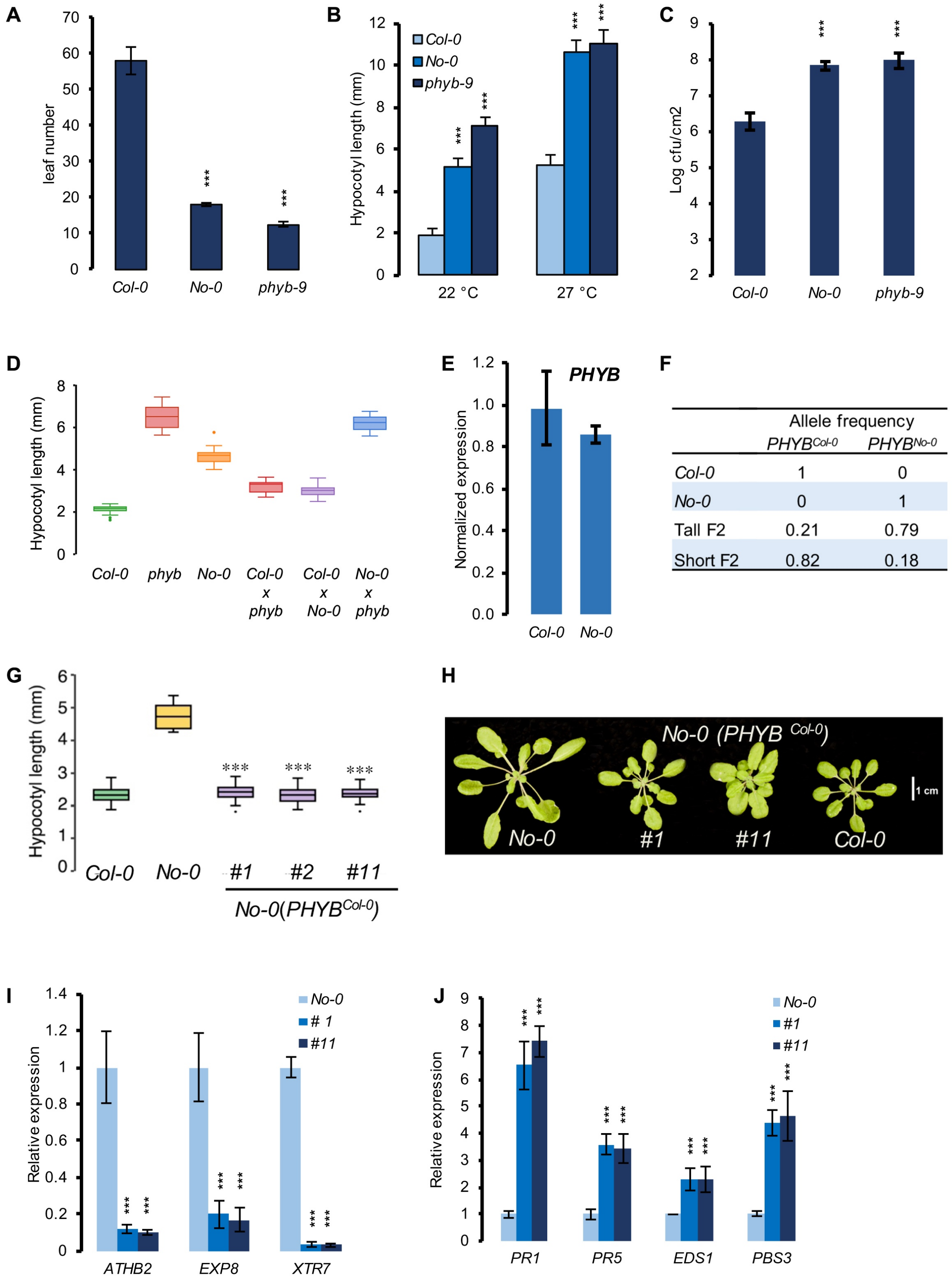

	Allele frequency	
	PHYB Col-0	PHYB No-0
Col-0	1	0
No-0	0	1
Tall F2	0.21	0.79
Short F2	0.82	0.18

Figure S2. ***PHYB* underlies altered growth-defense balance in No-0 (Related to Figure 3)** (**A**) Quantification of the flowering time data (mean±SD; n≥8) showing No-0 has early flowering phenotype similar to *phyb-9*. (**B**) Quantification of hypocotyl elongation data (mean±SD; n≥20) indicating that No-0 phenocopying *phyb-9* at different temperatures. (**C**) No-0 is increasingly susceptible to *Pto* DC3000 (A_{600}=0.02) at 22°C similar to *phyb-9* (data is mean±SD; n≥10). (**D**) Hypocotyl data of 22°C grown one-week-old seedlings showing *No-0* is allelic to *phyb* as $PHYB^{No\text{-}0}$ cannot complement *phyb-9* hypocotyl phenotype (Data shown is mean±SD; n≥20). (**E**) Expression of *PHYB* in No-0 is not altered as measured by qRT-PCR (mean±SD of three biological replicates) in one-week-old seedlings grown at 22°C SD. (**F**) Segregation analysis of No-0 × Col-0 F_2 population showing Tall hypocotyl phenotype is strongly associated with $PHYB^{No\text{-}0}$ (allele frequency= 0.79*)* whereas short hypocotyl phenotype is strongly associated with $PHYB^{Col\text{-}0}$ (with allelic frequency of 0.82). (**G**) Quantification of hypocotyl length (mean±SD; n≥20) of one-week-old seedlings grown at 22° C SD. ***P≤0.001 (Student's *t*-test) significantly different from No-0. (**H**) Rosette picture of four-week-old adult plants grown in 22° C SD showing complementation of No-0 by $PHYB^{Col\text{-}0}$. (**I** and **J**) Expression of growth (I) and defense (J) responsive genes (mean±SD of three biological replicates) in No-0 and two independent complemented (P_{PHYB}:$PHYB^{Col\text{-}0}$) lines from one-week-old seedlings. **P≤0.01, ***P≤0.001 (Student's *t*-test) significantly different from No-0.

In Figure A-C and F, data shown is representative of at least two independent experiments. ***P≤0.001, (Student's *t*-test) significantly different from Col-0.

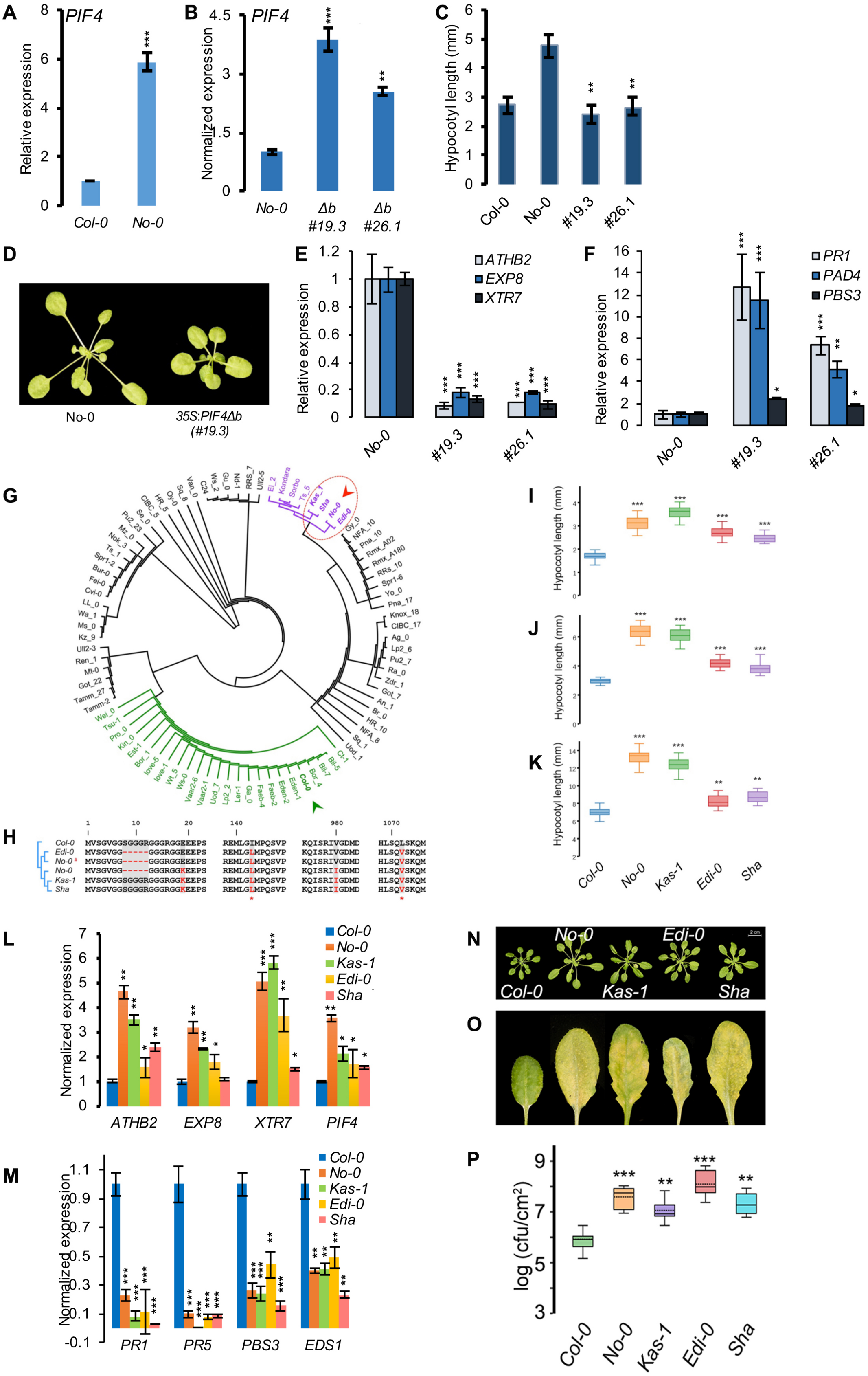

A
PIF4
Relative expression
Col-0
No-0
B
PIF4
Normalized expression
No-0
Δb #19.3
Δb #26.1
C
Hypocotyl length (mm)
Col-0
No-0
#19.3
#26.1
D
No-0
35S:PIF4Δb (#19.3)
E
ATHB2
EXP8
XTR7
Relative expression
No-0
#19.3
#26.1
F
PR1
PAD4
PBS3
Relative expression
No-0
#19.3
#26.1
G
H
I
J
K
Hypocotyl length (mm)
Col-0
No-0
Kas-1
Edi-0
Sha
L
Normalized expression
Col-0
No-0
Kas-1
Edi-0
Sha
ATHB2
EXP8
XTR7
PIF4
M
Normalized expression
Col-0
No-0
Kas-1
Edi-0
Sha
PR1
PR5
PBS3
EDS1
N
No-0
Edi-0
Col-0
Kas-1
Sha
O
P
log (cfu/cm²)
Col-0
No-0
Kas-1
Edi-0
Sha

Figure S3. Modulation of PIF4 function alters growth and defense in Arabidopsis natural accessions (Related to Figure 3)

(**A**) Expression of *PIF4* as measured by qRT-PCR (mean±SD of three biological replicates) in one-week-old seedlings grown at 22° C SD. ***P≤0.001 (Student's *t*-test) significantly different from Col-0 (**B**) Expression of total *PIF4* (*PIF4* + *PIF4Δb*) in two independent No-0(*35S:PIF4Δb-Myc*) transgenic lines (#19.3 and 26.1) as measured by qRT-PCR (mean±SD of three biological replicates). (**C**) Hypocotyl measurement data (mean±SD; n≥20) of No-0(*35S:PIF4Δb-Myc*) lines. (**D**) Rosette phenotype of No-0 overexpressing *PIF4Δb* showing reduced growth. (**E** and **F**) Expression of growth- (E) and defense responsive (F) genes as measured by qRT-PCR (mean±SD of three biological replicates). (**G**) Neighbour-joining tree of deduced PHYB amino acid sequence of the worldwide set of *Arabidopsis thaliana* natural accessions (obtained from 1001 genome project). Col-0 (green arrowhead) and No-0 (red arrowhead) are significantly divergent from each other. Edi-0, Kas-1, Sha and No-0 are highlighted to show their close similarity. (**H**) Alignment of deduced PHYB amino acid sequences of No-0, Kas-1, Edi-0, and Sha accessions in comparison to Col-0 showing conserved polymorphisms (red letters). (♯ indicates the laboratory accession of No-0). (**I-K**) Hypocotyl elongation of one-week old seedlings in No-0 related accessions at 17 (I), 22 (J) and 27ºC (K). (**L** and **M**) Upregulation of growth (L) and downregulation of defense responsive genes (M) in No-0-related ecotypes as measured by qRT-PCR (mean±SD of three biological replicates). (**N-P**) Natural accessions Kas-1, Edi-0, and Sha that show the similar amino acid sequence variations as No-0 show robust growth phenotypes (N) and enhanced disease susceptibility (O and P) phenocopying No-0.

In Figure A-C, E, F, and I-K, one-week-old seedlings grown at 22°C in SD were used for the experimental studies. *P≤0.05, **P≤0.01, ***P≤0.001 (Student's *t*-test) significantly different from either Col-0 (in I-M and P) or No-0 (in B, C, E and F).

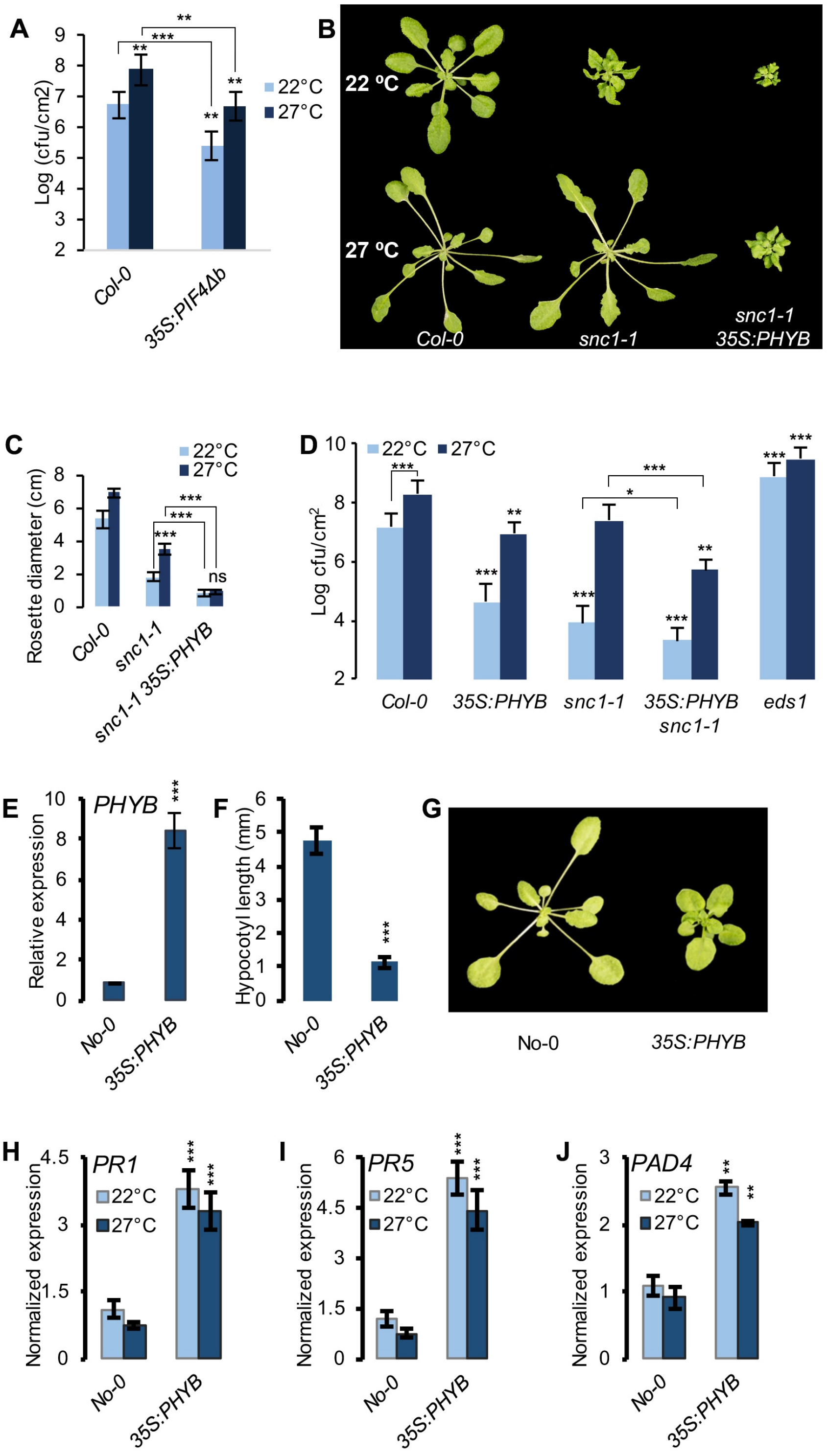

A
Log (cfu/cm2)
22°C
27°C
Col-0
35S:PIF4Δb
B
22 ºC
27 ºC
Col-0
snc1-1
snc1-1
35S:PHYB
C
Rosette diameter (cm)
22°C
27°C
Col-0
snc1-1
snc1-1 35S:PHYB
ns
D
Log cfu/cm2
22°C
27°C
Col-0
35S:PHYB
snc1-1
35S:PHYB
snc1-1
eds1
E
PHYB
Relative expression
No-0
35S:PHYB
F
Hypocotyl length (mm)
No-0
35S:PHYB
G
No-0
35S:PHYB
H
PR1
Normalized expression
22°C
27°C
No-0
35S:PHYB
I
PR5
Normalized expression
22°C
27°C
No-0
35S:PHYB
J
PAD4
Normalized expression
22°C
27°C
No-0
35S:PHYB

Figure S4. ***PHYB*** **overexpression leads to reduced growth and temperature resilient defense (Related to Figure 4)**

(**A**) Overexpression of *PIF4Δb* (*35S:PIF4Δb*) leads to temperature resilient disease resistance to *Pto* DC3000 (A_{600}=0.002) (mean±SD; n=8). **P≤0.01, ***P≤0.001 (Student's *t*-test) significantly different from Col-0. (**B**) Rosette phenotype of four-weeks old plants shows *35S*:*PHYB* enhances *snc1-1* phenotype at 22°C and prevents suppression by elevated temperature (27°C). **(C**) Quantification of rosette diameter (mean±SD; n≥8) data showing *35S:PHYB* can completely prevent *snc1-1* phenotype suppression by elevated temperature. (**D**) *35S:PHYB* suppresses elevated temperature mediated defense response of *snc1-1* to *Pto* DC3000 (A_{600}=0.02; data is mean±SD; n≥8). Four-week-old plants grown at SD were used for the assay. *P≤0.05, **P≤0.01, ***P≤0.001 (Student's *t*-test) significantly different from either Col-0 or between indicated pairs. (**E**) Expression of *PHYB* in *35S*:*PHYB* transgenic line in No-0 background as measured by qRT-PCR (mean±SD of three biological replicates) from one-week-old seedlings grown at 22° C SD. (**F**) Hypocotyl data (mean±SD; n≥20) of one-week-old seedlings grown at 22° C in SD. (**G**) Rosette phenotype of three-week-old plants grown at 22°C SD. (**H-J**) Expression of defense responsive genes in as measured by qRT-PCR (mean±SD of three biological replicates) from one-week-old seedlings grown at 22° and 27°C. **p≤0.01 and ***P≤0.001 (Student's *t*-test) significantly different from No-0.

Supplemental Experimental Procedures

Plant material and growth conditions: All experiments unless otherwise specified, were performed on *Arabidopsis thaliana* laboratory accession Col-0. Mutants *pif4-101*[S1], *snc1-1*[S2] and *phyb-9*[S3], and transgenic lines *35S:PHYB-GFP*[S4] and *35S:PIF4-HA*[S5] were previously described. The natural accessions No-0 (N3081), Kas-1(N22638), Edi-0 (N22657), and Sha (N22652) were obtained from The European Arabidopsis Stock Centre (Nottingham). The transgenic line *35S:PHYB*$^{Col\text{-}0}$ in No-0 (ABO) overexpressing *PHYB* has been described[S6]. For all the experiments, seeds were stratified for 3-days at 4 °C in the dark and germinated at 22 ºC short photoperiod (SD; 8h light/16 h dark photoperiod). Later they were either retained at 22 °C SD or transferred to 17 ºC or 27°C SD depending on the experiment.

Hypocotyl measurement: For hypocotyl measurement, seeds were surface sterilized and germinated at 22 ºC SD on ½ MS media. Two days post germination, seedlings were shifted to 17 ºC, 22 °C or 27 °C SD for seven days before they were aligned on 1% agar plate and imaged using stereomicroscope. At least 20 seedlings were used to measure hypocotyl length using NIH ImageJ software (http://rsbweb.nih.gov/ij/).

Generation of double mutants: For the generation of *snc1-1 pif4-101* double mutant, *snc1-1* was crossed to *pif4-101.* Genotyping for *pif4-101* (primers 85, 213 and 214) and *snc1-1* (primers 124 and 125) mutations to identify homozygous double mutants. In case of *scn1-1 PHYBOE*, *snc1-1* was crossed to *35S:PHYB-GFP* to get F1, and resulting F2 seeds were screened at 27 °C and seedlings with short hypocotyl were transferred to soil. Later, in adult stage these plants were genotyped for *snc1-1* mutation (primers 124 and 125), and in the next generation several plants with *snc1-1* mutation were screened on MS-kanamycin to get plants homozygous at the *35S:PHYB-GFP locus.*

Vector construction and generation of transgenic lines: Full length *PIF4* cDNA was amplified using oligo nucleotide primers 298 and 299 and cloned into pENTR/D-TOPO (Invitrogen) to generate *pENTR-PIF4*. Oligo nucleotide primers 301 and 302 were used to delete the basic domain of *PIF4* in *pENTR-PIF4* to get *pENTR-PIF4Δb*. Both full length *PIF4* and *PIF4Δb* were used to generate binary vectors *35S:PIF4-Myc* and *35S:PIF4Δb-Myc* respectively through recombination with the binary destination vector pGWB417[S7] using LR clonase (Invitrogen). Oligos 117 and 118 were used to amplify P_{PIF4}*:PIF4* genomic fragment containing native promoter region to generate pENTR-P_{PIF4}*:PIF4.* Binary vector *pPIF4:PIF4-FLAG* was generated through recombination with the binary destination vector

pKGW3F. *PHYB* gene fragment was amplified using oligo nucleotide primers 292 and 293 and was cloned into pENTR/D-TOPO to generate *pENTR-PHYB,* which was used to generate *35S:PHYB-FLAG* construct through recombination into pK35GW3F. Oligo nucleotide primers 896 and 293 were used to amplify P_{PHYB}*:PHYB* fragments before cloning into pENTR/D-TOPO to generate *pENTR-* P_{PHYB}*:PHYB*, which was recombined into pGWB610[S8] to generate the binary vector pP_{PHYB}*:PHYB.* All recombinant clones were validated through restriction analysis and were sequence confirmed for the junction and reading frame. The binary vector clones were transformed into *Agrobacterium* GV3101, and transformed into either Col-0 or No-0 using floral dip method. Transgenics were selecteed on either MS+Kanamycin (50 µg/ml; for *35S:PIF4Δb*, P_{PIF4}*:PIF4-FLAG* and *35S:PHYB-FLAG* lines) or MS+Basta (10 µg/ml; for P_{PHYB}*:PHYB*) to identify transgenics. In T1 generation, individual transgenic plants were screened for single copy transgene insertion using KASP marker (iDna Genetics). In the next generation, several plants from single copy transgenic lines were assayed to identify homozygous lines.

Pathogen assays: Bacterial infection assays using *Pseudomonas syringae pv. tomato* DC3000 (Pst DC3000) strain were done by spray inoculation of 4 week old plants either grown continuously at 22 ºC or shifted from 22 ºC to 27 ºC for three days before infection. Bacteria were adjusted to OD600 = 0.02 in 10 mM $MgCl_2$ with 0.04% Silwet L-77. Three days post inoculation, leaf discs from three leaves were collected, and bacteria was extracted by shaking the leaf dics in 10mM $MgCl_2$ with 0.01% Silwett L-77 at 28°C for 1 h. Bacterial titre was determined by plating a serial dilution of the suspension onto NYGA plates with Rifampicin (50µg/ml) and incubated for two days at 28°C. In all the experiments *eds1* was used as a positive control for susceptibility. Significant differences to the corresponding wild type were analyzed using Student's *t*-test or 2-way ANOVA analysis with Tukey's multiple comparison test as specified in the legends.

RNA expression analysis: For gene expression analysis using quantitative-PCR, RNA was extracted using RNeasy Plant mini kit (Qiagen) with on-column DNase I digestion according to the manufacturer's instructions). RNA was quantified using NanoDrop, and approximately 1.5 µg of total RNA was converted into cDNA using Superscript III reverse transcriptase (Invitrogen) and oligo *dT* according to the manufacturer instructions. cDNA was diluted 1:20 and 2.0µl was used for qPCR using 2X SYBR Greem Master Mix kit in Roche Lightcycler 480. Quantitative RT-PCR experiments were performed in Light Cycler LC480 using Light

Cycler 480 SYBR Green I Master (Roche). *Ef1α* (AT5G60390) was used as internal control for normalization. Details of the oligo nucleotide primers used are provided below. Primers were designed using the Primer3-BLAST (http://www.ncbi.nlm.nih.gov/tools/primer-blast/).

No-0 × Col-0 F2 co-segregation analysis: To determine the association of growth and disease resistance traits to the *PHYB* allelic variants, F2 seeds were sown onto soil for 10 days at 22 ºC SD, and at least 100 seedlings with short and long hypocotyls were identified and transferred to separate trays, and grown for a further three-weeks. These plants were assayed for resistance to *Pst* DC3000 along with parental controls (No-0 and Col-0) and *eds1* as positive control for susceptibility. All the plants were SNP genotyped for *PHYB* allele using KASP marker assay. Seedlings with long hypocotyls were co-segregated with $PHYB^{No\text{-}0}$ and disease susceptibility, whereas seedlings with short hypocotyls were co-segregated with $PHYB^{Col\text{-}0}$ and disease resistance.

RNAseq analysis: For RNA sequencing analysis total RNA was extracted from 10-day-old seedlings grown on ½ MS solid medium using the RNeasy Plant Mini Kit (Qiagen). Sequencing was performed at The Genome Analysis Centre using Illumina HiSeq 2500 using 50 bp single-end sequencing. TopHat v2.0.8 was used to align the reads to the Arabidopsis reference genome (TAIR 10). A differential expression analysis was run using Cuffdiff v2.0.2. Significance of expression change was determined based on the p-value corrected for multiple hypothesis testing and a false discovery rate of 0.05. Differential gene expression analysis was done on the RPKM values generated. Strand NGS was used to analyze differential gene expression and further detailed analysis of the RNA-seq data.

List of oligonucleotides used in this study

**Target**	**Oligo.**	**Oligo Sequence (5'…………..3')**	**Purpose**
P_{PIF4}:*PIF4-F*	117	*CACCGCCTGTTTAATTGGTGCTTGGTCAATTACG*	Native expression
P_{PIF4}:*PIF4-R*	118	*GTGGTCCAAACGAGAACCGTCGGTGGTCT*	Native expression
PHYB-F	292	*CACCAAACGGCATGGTTTCC*	Overexpression
PHYB-R	293	*ATATGGCATCATCAGCATCATGT*	Overexpression
PIF4-F	298	*CACCTTTCTGTCTGTACCCAAA*	Overexpression
PIF4-R	299	*GTGGTCCAAACGAGAACCGTCGG*	Overexpression
PIF4-Δb	301	GCAGCTGAAGTTGATAGGATCAATGAGAGAATG	Overexpression
PIF4-Δb	302	*ATTGATCCTATCAACTTCAGCTGCTCGACTCCTTC*	Overexpression
PHYB-F	896	*CACCCGAATAAGAGAAGGAATTAC*	Complementation
PHYB-R	293	*ATATGGCATCATCAGCATCATGT*	Complementation
PIF4-LP	213	*AATACATTTTGCAGGCAATCG*	Genotyping *pif4-101*
PIF4-RP	214	*CGTAATGAAGTTGCACGTTTACTC*	Genotyping *pif4-101*
PIF4-LB	85	*TTCATAACCAATCTCGATACAC*	Genotyping *pif4-101*
SNC1-FP	124	*ATACGTTTGCCATTCGAGGA*	Genotyping *snc1-1*
SNC1-RP	125	*ACCAGAGTTCCTTCCCACAG*	Genotyping *snc1-1*
PHYB-F	825	*CATTTGGCAATGCAGGGGAC*	Q-PCR
PHYB-R	826	*GTCCAACAAAACAAACGCCG*	Q-PCR
PIF4-F	766	*ACCTCAGAGACGGTTAAGCC*	Q-PCR
PIF4-R	767	*TGGAGGAGGCATGACTTGAG*	Q-PCR
ATHB2-F	667	*CCGTCGGCTACAAAAAGAAG*	Q-PCR
ATHB2-R	668	*GAAGGGCACATGGTCAAAGT*	Q-PCR
EXP8-F	633	*CTCTTTCCGAAGAGTACCATGT*	Q-PCR
EXP8-R	634	*GTGTACGTCTCCTGCTCCTC*	Q-PCR
XTR7-F	1127	*CGGCTTGCACAGCCTCTT*	Q-PCR
XTR7-R	1128	*TCGGTTGCCACTTGCAATT*	Q-PCR
PR1-F	18	*ACCAGGCACGAGGAGCGGTA*	Q-PCR
PR1-R	19	*TCCCCGTAAGGCCCACCAGA*	Q-PCR
PR5-F	20	*ACCCACAGCACAGAGACACACA*	Q-PCR
PR5-R	21	*TGGCCATAACAGCAATGCCGC*	Q-PCR
EDS1-F	140	*GAAGGGATGCTTGGAGAATG*	Q-PCR
EDS1-R	141	*AGTCTCGCAGAGGAGAATGC*	Q-PCR
PAD4-F	315	*TGGTGACGAAGAAGGAGGTT*	Q-PCR
PAD4-R	316	*TCCATTGCGTCACTCTCATC*	Q-PCR
PBS3-F	323	*TGAGTCAAGCGAAGCTCGTA*	Q-PCR
PBS3-R	324	*ATCGATCCGTCTTTGAATCG*	Q-PCR
SID2-F	351	*TCTCAATTGGCAGGGAGACT*	Q-PCR
SID2-R	352	*AAGCCTTGCTTCTTCTGCTG*	Q-PCR

Supplemental references

S1. Lorrain, S., Allen, T., Duek, P.D., Whitelam, G.C., and Fankhauser, C. (2008). Phytochrome-mediated inhibition of shade avoidance involves degradation of growth-promoting bHLH transcription factors. Plant J. *53*, 312–23.

S2. Zhang. Y., Goritschnig. S., Dong. X., and Li, X. (2003). A Gain-of-function mutation in a plant disease resistance gene leads to constitutive activation of downstream signal transduction pathways in suppressor of npr1-1, constitutive 1. Plant Cell *15*, 2636–46.

S3. Reed, J.W., Nagpal, P., Poole, D.S., Furuya, M., and Chory, J. (1993). Mutations in the gene for the red/far-red light receptor phytochrome B alter cell elongation and physiological responses throughout Arabidopsis development. Plant Cell *5*, 147–57.

S4. Ádám, É., Kircher, S., Liu, P., Mérai, Z., González-Schain, N., Hörner, M., Viczián, A., Monte, E., Sharrock, R.A., Schäfer, E., et al. (2013). Comparative functional analysis of full-length and N-terminal fragments of phytochrome C, D and E in red light-induced signaling. New Phytol. *200*, 86–96.

S5. Nozue, K., Covington, M.F., Duek, P.D., Lorrain, S., Fankhauser, C., Harmer, S.L., and Maloof, J.N. (2007). Rhythmic growth explained by coincidence between internal and external cues. Nature *448*, 358–61.

S6. Wagner, D., Tepperman, J.M., and Quail, P.H. (1991). Overexpression of phytochrome B induces a short hypocotyl phenotype in transgenic Arabidopsis. Plant Cell *3*, 1275–88.

S7. Nakagawa, T., Suzuki, T., Murata, S., Nakamura, S., Hino, T., Maeo, K., Tabata, R., Kawai, T., Tanaka, K., Niwa, Y., et al. (2007). Improved gateway binary vectors: high-performance vectors for creation of fusion constructs in transgenic analysis of plants. Biosci. Biotechnol. Biochem. *71*, 2095–100.

S8. Nakamura, S., Mano, S., Tanaka, Y., Ohnishi, M., Nakamori, C., Araki, M., Niwa, T., Nishimura, M., Kaminaka, H., Nakagawa, T., et al. (2014). Gateway binary vectors with the bialaphos resistance gene, bar, as a selection marker for plant transformation. Biosci. Biotechnol. Biochem. *74*, 1315–9.
